# Intra‐Operative Definition of Glioma Infiltrative Margins by Visualizing Immunosuppressive Tumor‐Associated Macrophages

**DOI:** 10.1002/advs.202304020

**Published:** 2023-08-06

**Authors:** Chong Cao, Hang Yin, Biao Yang, Qi Yue, Guoqing Wu, Meng Gu, Yuwen Zhang, Yang Fan, Xiaoyan Dong, Ting Wang, Cong Wang, Xiao Zhu, Ying Mao, Xiao‐Yong Zhang, Zuhai Lei, Cong Li

**Affiliations:** ^1^ Key Laboratory of Smart Drug Delivery Ministry of Education Innovative Center for New Drug Development of Immune Inflammatory Diseases, Ministry of Education School of Pharmacy Department of Neurosurgery, Huashan Hospital Fudan University Shanghai 201203 China; ^2^ State Key Laboratory of Medical Neurobiology Zhongshan Hospital Fudan University Shanghai 200032 China; ^3^ School of Information Science and Technology Fudan University Shanghai 200438 China; ^4^ Institute of Science and Technology for Brain‐Inspired Intelligence MOE Key Laboratory of Computational Neuroscience and Brain‐Inspired Intelligence MOE Frontiers Center for Brain Science Fudan University 220 Handan Road Shanghai 200433 China

**Keywords:** gliomas, hierarchical strategy, pH responsive probes, surgical navigations, tumor‐associated macrophages

## Abstract

Accurate delineation of glioma infiltrative margins remains a challenge due to the low density of cancer cells in these regions. Here, a hierarchical imaging strategy to define glioma margins by locating the immunosuppressive tumor‐associated macrophages (TAMs) is proposed. A pH ratiometric fluorescent probe CP2‐M that targets immunosuppressive TAMs by binding to mannose receptor (CD206) is developed, and it subsequently senses the acidic phagosomal lumen, resulting in a remarkable fluorescence enhancement. With assistance of CP2‐M, glioma xenografts in mouse models with a tumor‐to‐background ratio exceeding 3.0 for up to 6 h are successfully visualized. Furthermore, by intra‐operatively mapping the pH distribution of exposed tissue after craniotomy, the glioma allograft in rat models is precisely excised. The overall survival of rat models significantly surpasses that achieved using clinically employed fluorescent probes. This work presents a novel strategy for locating glioma margins, thereby improving surgical outcomes for tumors with infiltrative characteristics.

## Graphical abstract

A hierarchical imaging strategy is put forward in defining glioma infiltrative margin by visualizing immunosuppressive tumor‐associated macrophages instead of cancer cells. This strategy helps to unravel the challenge of defining the infiltrative tumor margins with sparse cancer cell distribution. This work is promising in reducing postoperative recurrence and prolonging patient survival.

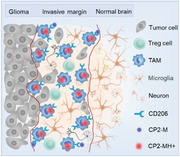

## 1 Introduction

Glioma accounts for ≈80% of all malignant brain tumors.^[^ ^1^ ^]^ Surgery has been the primary treatment approach for glioma. However, the prognosis of glioma patients heavily relies on the extent of tumor removal to prevent tumor residue recurrence. Unfortunately, the infiltrative nature of gliomas and the lack of techniques for visualizing the invasive margins result in a high recurrence rate after surgery.^[^ ^2^, ^3^, ^4^ ^]^ Hence, it is crucial to intraoperatively delineate the infiltrative regions of glioma to improve surgery prognosis.

Currently, the most widely used technology for guiding glioma surgery is stereotactic navigation, which relies on preoperative magnetic resonance imaging (MRI) to localize glioma margins.^[^ ^5^ ^]^ Nevertheless, the gadolinium‐based MR signal primarily depicts the region where the blood‐brain barrier (BBB) is disrupted, rather than the actual tumor infiltration.^[^ ^6^, ^7^ ^]^ Notably, intraoperative fluorescence imaging has attracted widespread attention due to its high sensitivity, wide field of view, and real‐time feedback.^[^ ^8^, ^9^ ^]^ Existing fluorescent probes approved for surgical navigation can be categorized into three types based on their imaging strategies. For example, indocyanine green (ICG), the most widely used near‐infrared (NIR) fluorescent agent in clinical settings, visualizes glioma through extravasation from the disrupted BBB.^[^ ^10^, ^11^ ^]^ However, its rapid excretion rate (*t*_1/2_≤ 5 min) and lack of tumor targeting specificity result in a low tumor‐to‐background (T/B) ratio.^[^ ^12^, ^13^ ^]^ Another example is 5‐aminolevulinic acid (5‐ALA), which can be selectively metabolized into photosensitizer protoporphyrin IX (PpIX) within cancer cells, exhibiting intense fluorescence upon excitation.^[^ ^14^ ^]^ However, it shows limited specificity in locating tumor‐infiltrated regions due to the false‐negative rate in low‐grade gliomas with attenuated metabolic activity.^[^ ^15^ ^]^ Even though above strategies offer clinical benefits, they encounter a common challenge in delineating tumor infiltrative regions due to the decreasing cancer cell density from the tumor to normal brain tissue. Consequently, the development of new strategies that outline tumor invasive margins without compromising cancer cell density is urgently needed.

Tumors represent spatially organized ecosystems comprising numerous cell types, each exhibiting a variety of phenotypes. Angelo et al. demonstrated the structural organization of the tumor‐immune microenvironment in terms of cellular composition, spatial arrangement, and regulatory‐protein expression.^[^ ^16^ ^]^ They introduced the concept of a “tumor immune boundary”, where ordered immune structures are found in tumor infiltrative margins and are associated with prognosis. Tumor‐associated macrophages (TAMs) are the most abundant immune cells in different types of solid tumors and are characterized by their plasticity and multiplicity.^[^ ^17^ ^]^ Boussioutas et al outlined the heterogeneity of TAMs in gastric cancer and revealed the disparity between TAMs subpopulations and intratumoral locations. They observed a prevalence of tumor‐promoting phenotypes (M2‐TAMs) at the margin, while a significant increase in the proportion of tumor‐suppressive TAMs (M1‐TAMs) was seen in the core. Notably, the population of M2‐TAMs increased from the core toward the infiltrative margin.^[^ ^17^ ^]^ Recent studies have reported the accumulation of TAMs around tumor foci in the early stages to promote cancer cell invasion.^[^ ^18^, ^19^ ^]^ Considering the spatial colocalization between M2‐TAMs and tumor infiltrative region, we hypothesize that M2‐TAMs could serve as an alternative marker to delineate glioma margins regardless of cancer cell density.

To establish the suppressive tumor immune microenvironment of glioma, TAMs polarize into different phenotypes with distinct metabolic pathways. The majority of TAMs in glioma are primed into M2‐TAMs, while a small subset adopts the M1‐TAM phenotype.^[^ ^20^, ^21^ ^]^ Notably, M1‐TAMs prefer energy metabolism via glycolysis and the pentose phosphate pathway, which activates NADPH oxidase 2 (NOX2) to produce reactive oxygen species (ROS). The dismutation of ROS into hydrogen peroxide consumes a large amount of protons, neutralizing (pH 7.2‒7.5) the phagosomal lumen.^[^ ^22^ ^]^ In contrast, M2‐TAMs depend on the oxidative phosphorylation pathway, leading to a more acidic phagosome (pH 4.5‒5.5).^[^ ^23^ ^]^ Therefore, identifying macrophage phenotypes by assessing phagosomal acidity holds promise.

By analyzing the specimens from glioblastoma (GBM) patients, we demonstrate the enrichment of M2‐TAMs in glioma‐invasive margins and establish a correlation between the number of M2‐TAMs and the prognosis of glioma patients. Accordingly, we propose a hierarchical imaging strategy to delineate glioma margins by targeting M2‐TAMs. We develop a pH ratiometric fluorescent probe, CP2‐M, that selectively targets CD206, a receptor specifically expressed on the M2‐TAMs. CP2‐M exhibits remarkable fluorescence enhancement in the acidic phagosomal lumen. In vivo and ex vivo fluorescence imaging demonstrates that the T/B ratio of CP2‐M is significantly higher than that of the control probes in mouse models bearing human glioma xenograft. Furthermore, CP2‐M enables the intra‐operative localization of intracranial glioma allograft in rat models, maintaining a T/B ratio above 3.0 for more than 6 h. In comparison to 5‐ALA or ICG‐guided surgery, CP2‐M‐guided surgery significantly reduces tumor relapse and improves behavioral performances in animal models. Overall, this hierarchical imaging strategy provides an alternative approach to defining glioma margins and enhancing surgical prognosis by visualizing the M2‐TAMs.

## 2 Results

### 2.1 M2‐TAMs Abundancy is Associated with Glioma Malignancy and Poor Prognosis

**Figure 1:**
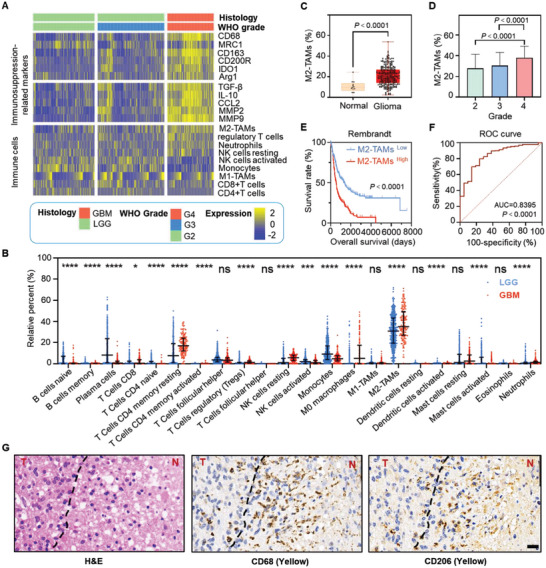
M2‐TAMs accumulate at the invasive margins of GBM. A) The heatmap shows the immune profiles in the TCGA‐glioma cohort. The upper panel demonstrates the expression of biomarkers involved in immunosuppression. The below panel demonstrates the densities of immune cells in glioma. The histology and WHO grade of glioma are annotated at the top of the heatmap. B) The relative percentage of immune cells in LGG (blue plot) versus GBM (red plot) was analyzed in the TCGA database. The scattered dot represents the relative percentage of immune cells in the tumor sample from each glioma patient. C) The percentages of M2‐TAMs in normal brain and glioma tissues from patients. D) The percentages of M2‐TAMs as a function of glioma grades. E) Kaplan‐Meier survival curves comparing overall survival between glioma patients with low (blue) and high (red) percentages of M2‐TAMs in the Rembrandt database. Tick marks indicate censoring. F) Receiver‐operating characteristic (ROC) curve for prediction of diagnosis in patients with glioma. When AUC > 0.7, gliomas were accurately diagnosed by the percentages of M2‐TAMs. G) Representative H&E, CD68, and CD206 immunohistochemistry images of marginal tumor region from the adjacent slices of patient glioma sample. Scale bar: 20 µm. T: tumor; N: normal tissue; LGG: low‐grade glioma; GBM: glioblastoma; ROC: Receiver operating characteristic; AUC: The area under curve; OS: overall survival. NS  =  non‐significant, *p < 0.05, ***p < 0.001, ****p < 0.0001. Data are given as the mean ± SD., the statistical significance of the survival curve was calculated using the log‐rank test in E, and a two‐tailed Student's t‐test was performed in C, D, and F. Statistical significance was considered for a *p*‐value of <0.05.

To identify reliable indicators that could accurately delineate the infiltrative margins of glioma, we analyzed RNA‐sequence data from The Cancer Genome Atlas (TCGA)‐glioma project consisting of low‐grade glioma (LGG) and high‐grade glioblastoma (GBM) specimens. In terms of clinical characteristics (from 698 glioma patients), we depicted a comprehensive heatmap of immunosuppression‐related markers, immune cells, and their relationship to glioma malignancy (**Figure** **1**A). Compared to LGG (including World Health Organization (WHO) Grade 2 and 3), the GBM (WHO Grade 4) specimens demonstrated an elevated number of immunosuppressive cells including regulatory T cells, resting natural killer (NK) cells, and M2‐TAMs in particular. Moreover, immunosuppression‐related markers, including Arginine 1 (Arg 1), transforming growth factor‐β (TGF‐β), interleukin (IL‐10), matrix metalloprotein 9 (MMP 9), and M2‐TAMs‐related receptors (CD68, CD206, and CD163), were increased with WHO grade. We further compared the percentage of 22 immune cell subtypes between LGG and GBM specimens in TCGA (Figure 1B), M2‐TAMs were found with the highest proportion among the immune cells and had a higher composition in GBM than that in LGG (*p* < 0.0001). In addition, the proportion of M2‐Mφ in the tumors was significantly higher than that in normal brain tissue from the Repository of Molecular Brain Neoplasia Data (Rembrandt) (*p* < 0.0001, Figure 1C). Meanwhile, the proportion of M2‐TAMs was also increased with WHO grade (Figure 1D). Simultaneously, survival curves uncovered that the patients with a higher density of M2‐TAMs had a shorter overall survival probability than those with a lower density (Figure 1E, S1). Furthermore, the receiver operating characteristic (ROC) analysis showed a good performance of M2‐TAMs in glioma diagnosis with the area under the curve (AUC) of 0.8395, meaning that M2‐TAMs are clinically valuable for predicting glioma (Figure 1F). Western blot showed that CD206 was highly expressed in M2‐like macrophages (M2‐Mφ) compared to other phenotypes (Figure S2, Supporting Information). Immunohistochemical staining of the glioma tissue from GBM patients revealed that CD68^+^ CD206^+^ M2‐TAMs (mainly referred to as M2‐Mφ) were enriched at the infiltrative margins of glioma (Figure 1G). The above investigations imply that M2‐TAMs could be a valuable marker of glioma infiltrative margins.

### 2.2 Design and Characterization of Aiming Probe CP2‐M

**Figure 2:**
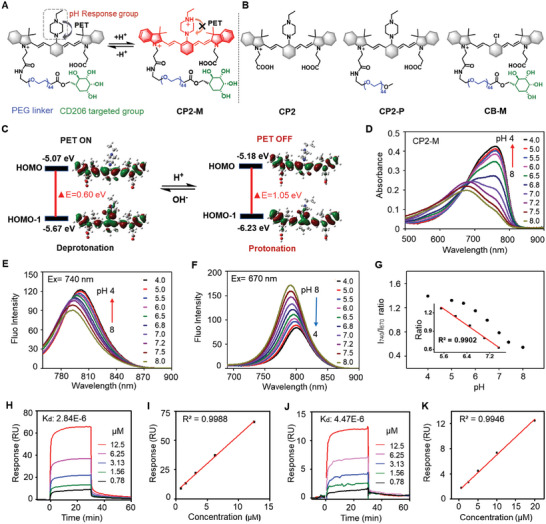
Synthesis and characterization of CP2‐M. A) Chemical structures of CP2‐M in its protonated and deprotonated forms. B) Chemical structures of control probes including CP2, CP2‐P modified with PEG2K, and CB‐M modified with PEG2K and mannose but without pH responsiveness. C) The HOMO and HOMO‐1 energy levels for CP2, based on density functional theory (DFT) calculations at the B3LYP/6–311 G (d,p) level. Absorption D) and emission spectra E,F) of CP2‐M as a function of pH. CP2‐M was excited at 740 and 670 nm, respectively. G) Plotting fluorescence intensity ratio (I740/I670 nm) as a function of pH. Inset: the linear relationship between I740/I670 ratio and pH value. H−K) Surface plasmon resonance (SPR) H,I) binding analysis and concentration‐response plots of PEG2K‐mannose and J,K) CP2‐M binding CD206 performed at concentrations between 0.78 µM and 12.5 µM with fitted affinity (1:1 steady‐state affinity model). Steady‐state binding curves from SPR indicate that the binding affinity depends on the concentration of I) PEG2K‐mannose and K) CP2‐M.

To define glioma infiltrating borders, we design a hierarchical probe CP2‐M by first targeting CD206 and then responding to phagosome acidity in M2‐TAMs.^[^ ^24^ ^]^ **Figure** **2**A shows the chemical structure of aiming probe CP2‐M in its protonated and deprotonated forms. CP2‐M consists of an ethyl‐piperazine‐substituted IR783 fluorophore and a targeting ligand‐mannose covalently coupled via a polyethylene glycol 2000 (PEG_2k_) linker. Figure 2B presents the chemical structures of control probes including CP2, a pH‐responsive small molecule fluorophore, CP2‐P in which a pH‐responsive fluorophore is modified with a PEG_2k_ moiety, CB‐M in which a pH‐inert fluorophore is modified with a targeting ligand mannose. The NIR fluorescence of CP2‐M is quenched due to the photo‐induced electron transfer(PeT)effect between IR783 and piperazine. After being protonated, the PeT process was inhibited, resulting in the recovery of the fluorescence of CP2‐M centered at 800 nm. This hypothesis was also verified via the descriptor Δ*E*energy gap between the Highest Occupied Molecular Orbital (HOMO) and HOMO‐1as a predictor of low/high quantum yields proposed by Liu et al.^[^ ^25^ ^]^ According to the theory, the PeT process is significantly suppressed when Δ*E* > ≈0.6 eV, which results in high quantum yields. Meanwhile, Δ*E* < ≈0.6 eV leads to a fast PET process and fluorescence quenching. As expected, quantum chemical calculations showed that Δ*E* of CP2 in the protonated state (1.05 eV) was significantly larger than 0.6 eV (Figure 2C), while Δ*E* of CP2 in the non‐protonated state was 0.6 eV. As expected, quantum chemical calculations validated the rational design of the CP2‐M response to pH in terms of the theoretical model.

The pH‐dependent absorption/emission spectra of control probes CP2, CP2‐P, and CB‐M were collected (Figure S3, Supporting Information).^[^ ^26^ ^]^ CP2‐M displayed pH‐responsive absorption and emission spectra (Figure 2D‒F). On decreasing the pH value, the maximum absorption peak of CP2‐M at ≈670 nm significantly diminished, and a new red‐shifted absorption peak at ≈740 nm was simultaneously formed. These findings suggested that when protonation occurs at the two nitrogen atoms of piperazine, it will lead to a red‐shift of the absorption peak owing to the decrease of the electron‐donating ability of the amine of CP2‐M. Accordingly, the intensity of fluorescence excited by 670 nm decreases with acidification, while the one excited by 740 nm was just the opposite, and their maximum emission wavelengths show a red‐shifted with acidification (Figure 2E‒F, S4). Thus, the ratio of fluorescence intensities at 740 and 670 nm (*I*_740_/*I*_670_) gradually increased and varied from pH 8.0 to pH 4.0 (Figure 2G). Furthermore, the fluorescence intensity ratios (*I*_740_/*I*_670_) and pH value in the range 5.5‒7.5 showed excellent linearity with a liner‐regression equation *I*_740_/*I*_670_ = −0.3421 × pH + 3.183 (*R* ^2^ = 0.9902), indicating that CP2‐M can function as a dual‐excitation ratiometric fluorescent pH probe to quantitatively determine pH values. Remarkably, the fluorescence intensity ratio *(I*_740_ */I*_670_) of CP2‐M (5.0 µM) showed photophysical stability and high selectivity for sensing pH variation (Figures S5  S6, and S7, Supporting Information). In addition, CP2‐M could reversibly monitor pH variation in the physiological range even after four switching cycles from pH 4.0‒7.4 (Figure S8, Supporting Information). We further conducted surface plasmon resonance (SPR) analysis to measure the binding affinity between the CP2‐M and mannose receptor (CD206) protein at pH 7.4. The Kd values of 2.84 µM for PEG_2K_‐mannose (Figure 2H,I) and 4.47 µM for CP2‐M (Figure 2J,K) were measured, indicating that the mannose conjugated in CP2‐M retains its binding affinity to CD206. The above results verify that CP2‐M has both pH responsiveness and binding affinity to CD206.

### 2.3 CP2‐M Visualizes Anti‐Inflammatory Macrophages In Vitro

**Figure 3:**
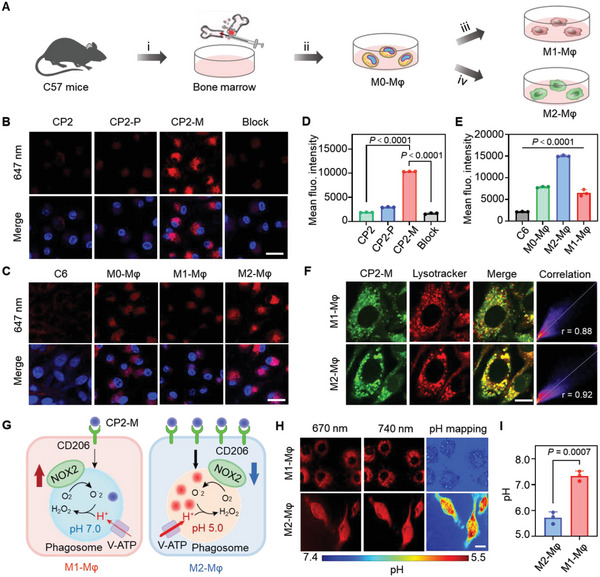
CP2‐M identifies M2‐Mφ with high specificity. A) Isolation, differentiation, and polarization of primary macrophages from mouse bone marrow. i) extraction of bone marrow, ii) differentiation into M0‐Mφ by M‐CSF, iii) polarization into M1‐Mφ via LPS/IFN‐γ, and iv) into M2‐Mφ via IL‐4. B) Confocal fluorescence microscope images of live M2‐MΦ after treatment with CP2, CP2‐P, or CP2‐M in the presence and absence of mannose (blockade control); C) Confocal fluorescence images of live C6 GBM cancer cells, M0‐Mφ, M1‐Mφ, and M2‐Mφ treated with CP2‐M (25.0 µM) for 1 h, scale bar: 30 µm; D,E) Quantified intracellular mean fluorescence intensities (MFI) of CP2‐M by flow cytometry statistical analysis corresponding panel B and panel C; F) Fluorescence images of macrophages treated with CP2‐M and Lysotracker, respectively. Yellow regions indicated the CP2‐M uptake in the lysosomes, scale bar: 10 µm; G) Illustration of CP2‐M specifically visualizing M2‐MΦ instead of M1‐MΦ or cancer cells via hierarchical strategy; H) pH map of live macrophages after treatment of CP2‐M (25.0 µM). pH maps were generated from the ratiometric fluorescence signal excited at 670 and 740 nm, *λ*_em_  =  780−830 nm. Scale bar: 10 µm. I) Average intracellular pH values measured in the phagosomes of M1‐Mφ and M2‐Mφ. Mφ: macrophages. Data with error bars are expressed as mean ± S.D. (*n*  =  3), and a two‐tailed unpaired t‐test is used for comparison between two sets of data in D, E, and I. Statistical significance was considered for a *p*‐value of < 0.05.

The hierarchical targeting capability of probe CP2‐M was evaluated in the bone marrow‐derived macrophage culture system. Briefly, bone marrow was removed from C57 mice and cultured with macrophage colony‐stimulating factor (M‐CSF) into primary macrophages, which were then polarized to anti‐inflammatory phenotypes (M2‐Mφ) in presence of IL‐4 or pro‐inflammatory phenotypes (M1‐Mφ) in presence of lipopolysaccharides (LPS) and IFN‐γ (**Figure** **3**A). Low cytotoxicity of CP2‐M was observed after culturing with M2‐Mφ for 48 h (Figure S9, Supporting Information). Flow cytometric analysis revealed that the uptake of CP2‐M reached an equilibrium in M2‐Mφ after culturing for 2 h (Figure S10, Supporting Information). After a 2 h incubation between probe (CP2, CP2‐P or CP2‐M) and M2‐Mφ, more than 4‐fold stronger fluorescence signals were observed in CP2‐M group than in other groups (Figure 3B,D). Furthermore, the fluorescence signal was significantly weakened if CD206 was blocked with mannose in advance, suggesting that CP2‐M is uptake by M2‐Mφ via CD206‐mediated endocytosis. After co‐incubation of CP2‐M with GBM cancer cells (C6) or macrophages with different phenotypes, the highest fluorescence signal of CP2‐M was observed in M2‐Mφ (Figure 3C,E). Similar results were observed for human‐derived GBM tumor cells (U251) and human‐derived macrophages with different phenotypes (Figure S12, Supporting Information). Since M2 microglia also highly express CD206 receptors, CP2‐M has high uptake in them as well. To investigate the intracellular trafficking of CP2‐M, we incubated polarized macrophages with Lysotracker Green DND‐26. Pearson correlation coefficients of 0.88 in M1‐Mφ and 0.92 in M2‐Mφ were measured respectively (Figure 3F), which indicates the specific uptake of CP2‐M into lysosomes. To further confirm the pH responsiveness of CP2‐M in the cytoplasm, we used the buffered solutions with varying pHs to equilibrate the intracellular pH. A decrease in fluorescence intensity was observed upon acidification, and the average signal intensity of 633 nm channel at pH 7.4 is 1.8 times higher than that at pH 5.5 (Figure S11, Supporting Information). Figure 3G showed a schematic illustration of CP2‐M visualizing M2‐Mφ via hierarchical imaging strategy. CD206, specifically expressed on the cell membrane of M2‐Mφ, mediates the selective endocytosis of CP2‐M into M2‐Mφ rather than cancer cells or M1‐Mφ. The phenotype‐dependent metabolic pathway leads to the distinct acidities of the lysosomal lumen in macrophages. Thus, the pH value of phagosomes could be regarded as a specific indicator to further distinguish M2‐Mφ from M1‐Mφ. The pH maps converted from fluorescence intensity ratio (*I*_740_/*I*_670_) showed that the average cytoplastic pH of M1‐Mφ was determined as 7.2, while the pH value of M2‐Mφ was measured as 5.7 (Figure 3H,I). The above cellular experiments confirm that CP2‐M could visualize M2‐Mφ with high specificity and sensitivity.

### 2.4 CP2‐M Visualizes Glioma with Improved Pharmacokinetics

**Figure 4:**
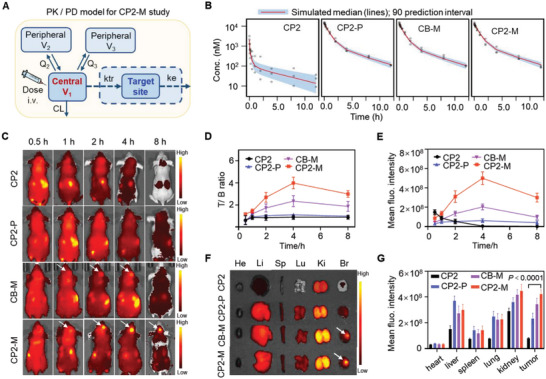
CP2‐M locates GBM xenograft with high specificity. A) Schematic of the PK/PD model investigating fluorescent probes, which includes a three‐compartment PK model along with an effect compartment for the target site of the probes. B) A visual predictive check from the PK model shows that the simulated probe concentrations have the same trend and variation as the observed data. The gray dots are the experimental data, the solid red line indicates the predicted 50 percentile, and the blue area represents the 90% prediction interval of the final model simulation. C) In vivo fluorescence images of mouse models at selected time points after intravenous injection of CP2, CP2‐P, CB‐M, or CP2‐M (with CP2 dose of 5.0 µmol kg^−1^). The probes were excited at 740 nm and emission between 780‒820 nm was collected. Tumors are indicated by D) white arrows. T/B ratio and E) mean fluorescence intensity at tumor site as a function of time post probe administration. F) Fluorescence images of heart (He), liver (Li), spleen (Sp), lung (Lu), kidney (Ki), and brain (Br) from mouse models sacrificed at 8 h post‐injection of CP2, CP2‐P, CB‐M or CP2‐M (5.0 µmol kg^−1^). G) Quantitative analysis of mean fluorescence intensity in the excised tumors and major organs. Data are given as the mean ± SD (*n* = 3 mice per group), and the two‐way analysis of variance (ANOVA) was performed in panel G. Statistical significance was considered for a *p*‐value of < 0.05.

We further investigated the pharmacokinetics (PK) and pharmacodynamics (PD) of this probe in rats. To better determine the PK and PD differences among the PEG_2K_‐modified probes (CP2‐M, CB‐M, and CP2‐P) and small molecule probe CP2 in rats, a three‐compartment model was developed based on the results of goodness‐of‐fit (GOF) plots and Akaike information criterion (AIC) (**Figure** **4**A). The plasma concentration‐time curve showed PK profiles of the probes over 12 h after an administration (5.0 µmol kg^−1^) via tail vein injection (Figure 4B). While the half‐life (*t*_1/2_) of CP2 was determined as 0.04 ± 0.003 h, the corresponding values of CP2‐M, CB‐M, and CP2‐P were measured as 1.11 ± 0.07 h (*n* = 3), 1.07 ± 0.04 h (*n* = 3) and 1.04 ± 0.06 h (*n* = 3). All PK parameters were precisely estimated with a relative standard error (RSE) of less than 40%. The visual predictive check (VPC) (Figure 4B) results suggested that the final model described the general trend of the experimental data well and adequately captured the variability in this animal study. The clearance of PEG_2K_‐modified probes (CP2‐M, CB‐M, and CP2‐P) profoundly decreased to 6.5% of small molecule probe CP2 (1.13 to 0.0732 L h^−1^), and the inter‐compartmental clearance also decreased to within 2.8% of CP2 (Q2: 2.26 to 0.0295 L h^−1^, Q3: 4.92 to 0.139 L h^−1^) (**Table** **1**). Similarly, the modification of PEG also resulted in a substantial decrease in the peripheral compartment distribution volume of probes (V2: 4.39 to 0.097 L, V3: 0.422 to 0.0113 L), whereas the central compartment distribution volume was comparable to that of PEG‐free probe (0.0515 vs 0.0756 L) (Table 1). All these findings indicate the profoundly extended system circulation time and substantially reduced permeability of the probes after the modification of PEG. In addition, the modification of the mannose had no significant effect on the PK behaviors of probes in the rat plasma.

**Table 1:** The parameters of the probes’ PK/PD model

	CP2	CP2‐P and CB‐M and CP2‐M		
PK Parameters	Estimation [RSE%^a^]	Estimation [RSE%]		
CL, L h^−1^	1.13 (37%)	0.0732 (5%)		
V_1_, L	0.0515 (8%)	0.0756 (4%)		
V_2_, L	4.39 (12%)	0.097 (10%)		
Q_2_, L h^−1^	2.26 (23%)	0.0295 (9%)		
V_3_, L	0.422 (13%)	0.0113 (18%)		
Q_3_, L h^−1^	4.92 (5%)	0.139 (31%)		
PD Parameters	CP2	CP2‐P	CB‐M	CP2‐M
ktr h^−1^	–	0.76 (4%)	0.666 (9%)	0.719 (5%)
ke h^−1^	–	1.15 (4%)	0.703 (2%)	0.475 (1%)
ktr/ke	0.931 (3%)	–	–	
Residual error	Estimation (RSE%)	Estimation (RSE%)		
Prop.RE(PK)	44.3% (30%)	27.6% (10%)		
Add.RE(PK)	4.07 (46%)	27.2 (26%)		
Prop.RE(PD)	34.8% (5%)	51.7% (9%)		
Add.RE(PD)	0.0044 (2%)	–		
Between‐subject variability	Estimation (RSE)[SHR]^b)^	Estimation (RSE)[SHR]		
CL	69.1% (22%)[0%]	13.2% (17%)[0%]		
V_2_	65.1% (36%)[0%]	25.6% (14%)[11%]		
Q_2_	43.7% (27%)[0%]	15.6% (26%)[21%]		

^a)^ RSE: relative standard error

^b)^ SHR: shrinkage

CL: clearance; V_1_: volume of the central compartment; V_2_: volume of the first peripheral compartment; Q_2_: the inter‐compartmental clearances between central and the first peripheral compartment; V_3_: volume of the second peripheral compartment; Q_3_: the inter‐compartmental clearances between central and the second peripheral compartment; ktr: the rate constant of drug flow from the central compartment to the target compartment; ke: elimination rate constant from the target compartment.

The time‐course of whole‐body fluorescence imaging was performed in nude mouse models bearing orthotopic human U251 glioma xenograft. As shown in the Figure 4C, no marked fluorescence signal was observed at the tumor site after intravenous injection with CP2 or CP2‐P, while, two mannose‐modified probes (CB‐M and CP2‐M) could indicate the boundaries of tumors at 1‒4 h post administration. More importantly, CP2‐M showed a significantly higher T/B ratio (Figure 4D) and MFI (Figure 4E) at tumor site when compared to other probes, and its high T/B ratio could last for >8 h. Furthermore, the prolonged resident time of PEG‐modified probes was also confirmed via ex vivo fluorescence images of the isolated organs from mouse models at 4 h post‐administration (Figure 4F). The fluorescence intensity of CP2‐M in tumor region was 5.3, 1.8, and 1.2‐fold higher than that of CP2, CP2‐P, and CB‐M (Figure 4G), further demonstrating its tumor specificity. Frozen sections were made from rat brain tissue at 4 h after probe injection into the body. Confocal microscopy images showed that CP2‐M were clustered in the tumor area and there was little CP2‐M signal in normal tissue (Figure S13, Supporting Information). Moreover, the CP2‐M signal was well co‐localized with CD206 (Figure S14, Supporting Information). These findings supported the superiority of CP2‐M locating tumors via the hierarchical strategy.

**Figure 5:**
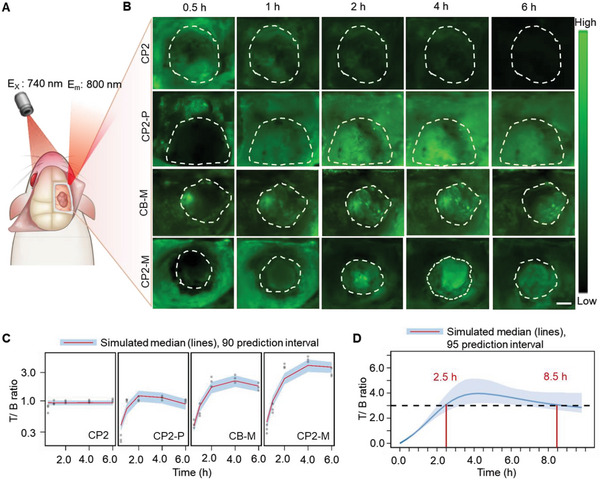
CP2‐M defines glioma allograft in rat models after craniotomy. A) Schematic diagram of in vivo imaging intracranial glioblastoma xenograft via 740 nm excitation wavelength. B) Intra‐operative fluorescence images of intracranial tumor region at selected time points after intravenous injection of CP2, CP2‐P, CB‐M, or CP2‐M (5.0 µmol kg^−1^). Tumors are delineated with white dashed lines, scale bar: 2.0 mm. C) A visual predictive check from the final PD model shows that the simulated T/B ratios have the same trend and variation as the experimental data. D) Time‐dependent T/B ratios after a single administration of CP2‐M (5.0 µmol kg^−1^). The black dashed line indicates the ideal T/B value and the time slot between the two red lines is the recommended time window for operation.

The capabilities of CP2, CP2‐P, CB‐M, and CP2‐M to delineate glioma margins were investigated after the injection of the probes into rat models bearing C6 murine glioma allograft. In vivo fluorescence images of the rat brain were collected after irradiating with a 740 nm laser at selected time points post‐probe administration (**Figure** **5**A). The fluorescence intensity of CP2 in the brain reached its maximum at 30 min but decreased rapidly, while much brighter fluorescence signal of CP2‐P, CB‐M, and CP2‐M in tumor region lasted for more than 4 h. Importantly, the fluorescent signals of CB‐M and CP2‐M increased specifically in tumors but decreased in surrounding normal brain tissue (Figure 5B). Notably, the T/B ratio of CP2‐M reached its peak value of 4.94 at 4 h post‐administration, while the value of CP2‐P fluctuated ≈1.0 (Figure 5C).

All the pharmacodynamic parameters were estimated with RSE < 30% (Table 1). As a small molecule probe, CP2 demonstrated high permeability and instantaneously reached an equilibrium between the target site and plasma after the injection, with an equilibrium constant close to 1 (i.e., no signal accumulation compared to the noise). In contrast, PEG modification resulted in the significantly slower distribution of CP2‐P, CB‐M, and CP2‐M (ktr of CP2‐P, CB‐M, and CP2‐M: 0.760, 0.666, and 0.719 h^−1^). In addition, mannose modification enhanced the targeting specificity of CB‐M and CP2‐M to CD206, leading to a decrease in its elimination from the tumor (ke of CP2‐P: 1.15 h^−1^; ke of CB‐M and CP2‐M: 0.703 and 0.475 h^−1^) and prolonged resident time of T/B > 3 (Figure 5C). Furthermore, the simulation from the final PK/PD model illustrated that the optimal imaging window was between 2.5 and 8.5 h post‐injection of CP2‐M (15 mg kg^−1^) (Figure 5D).

### 2.5 CP2‐M Defines Glioma Infiltrative Margins

**Figure 6:**
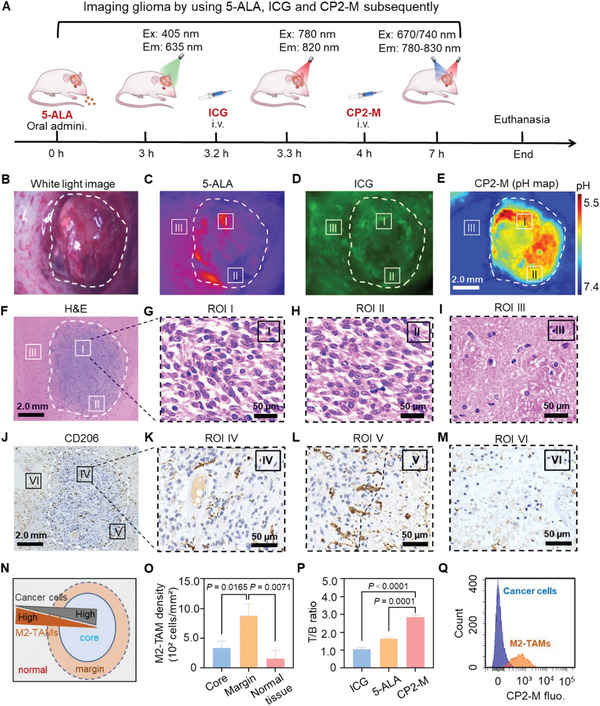
CP2‐M locates GBM margins by targeting M2‐TAMs. A) Flow chart presents in vivo fluorescence imaging of GBM allograft in the same rat model by using three fluorescent probes subsequently. B) C) Representative white light, fluorescence images of tumor region post oral administration of 5‐ALA, D) intravenous injection of ICG, E) pH map delineated by intravenous administrated CP2‐M. The pH map of the tumor region was generated by determining the ratio of CP2‐M fluorescence intensities after excitation at 740 and 670 nm, respectively. The dotted lines indicate the tumor body. The color bar represents the pH magnitude. Scale bars, 2.0 mm. F) Representative H&E staining image of glioma xenograft (B) and the enlarged images of the region G) I, H) II, and I) III, scale bars: 2.0 mm and 50 µm (enlarged). J) Immunohistological staining of CD206+ TAMs in tumor and the surrounding region. Spatial distribution of CD206+TAMs in regions K) IV, L) V, and M) VI. N) Schematic illustration presenting spatial distribution patterns of M2‐TAMs and cancer cells in glioma. O) M2‐TAMs densities in different regions of the tumor. P) The maximal T/B ratio of the probes measured to visualize GBM allograft. Q) Flow cytometric analysis of intracellular fluorescence intensity of cancer cells and M2‐TAMs isolated from tumor tissue at 4 h post‐CP2‐M administration. scale bars: 2.0 mm and 50 µm (enlarged). Data with error bars are expressed as mean ± S.D. (*n*  =  3), and an unpaired t‐test was used for comparison between two sets of data in O, and P. Statistical significance was considered for a *p* value of <0.05.

We evaluated the capability of CP2‐M to visualize orthotopic glioma allograft in comparison to the clinically used ICG and 5‐ALA in the same rat model bearing GBM allograft (**Figure** **6**A). Figure 6B showed a white light image of exposed GBM allograft after craniotomy. Red fluorescence induced by 5‐ALA was distributed heterogeneously in the tumor and the negative signal was indeed observed in part of the tumor region (Figure 6C). After the vanishment of 5‐ALA signal in the tumor bed, ICG (0.2 µmol) was intravenously injected into the same rat model. Non‐specific fluorescence signal of ICG was detected in both the tumor and surrounding normal brain tissues. The compromised T/B ratio resulted in the incapability of ICG to distinguish GBM margins (Figure 6D). Next, the pH map of tumor cutting edges was delineated intra‐operatively by fitting the fluorescence intensity ratio (I_740_/I_670_) of CP2‐M at each pixel with the calibration curve. Notably, the pH map revealed the acidification of the whole tumor including the negative regions indicated by 5‐ALA (Figure 6E). At the end of imaging, this brain tumor was sectioned and stained for pathological validation (Figure 6F). H&E staining showed that the region with high acidity (pH < 6.0, ROI I) indicated by CP2‐M was highly malignant (Figure 6G). Notably, the region (ROI II) with the negative signal of 5‐ALA but the acidic signal of CP2‐M was verified as the malignant tissues (Figure 6H), which suggests the reliability of CP2‐M to define the tumor‐infiltrating areas. Significantly, the tumor neighboring region (ROI III) with the positive signal of 5‐ALA but neutral pH measured by CP2‐M was confirmed to be normal brain tissue (Figure 6I). Immunohistochemical staining showed the up‐regulated CD206^+^ TAMs density within tumor tissues (Figure 6J). In contrast to the tumor core region (ROI IV, Figure 6K), the CD206^+^ TAMs prefer to accumulate at glioma margins as multiple cluster structures (ROI V, Figure 6L). Notably, the scatteringly distributed CD206^+^ TAMs were also observed in tumor‐neighboring brain tissues (ROI VI, Figure 6M). Figure 6N demonstrates the reversed gradients of cancer cell and M2‐TAMs (CD206^+^) densities across the tumor and neighboring brain tissues. Notably, the trend was observed that the densities of M2‐TAMs decreased from the tumor margin toward the core (Figure 6O). Overall, CP2‐M more comprehensively delineated tumor‐infiltrating margins with a higher T/B ratio in comparison to that of 5‐ALA and ICG (Figure 6P). Flow cytometric analysis of the cells isolated from brain tumor tissues at 4 h post‐CP2‐M administration showed that the intracellular fluorescence intensity of M2‐TAMs was much higher than that of cancer cells (Figure 6Q). Notably, very few M1‐TAMs were collected from the brain tissue.

### 2.6 CP2‐M Guided Glioma Surgery in Rat Models

**Figure 7:**
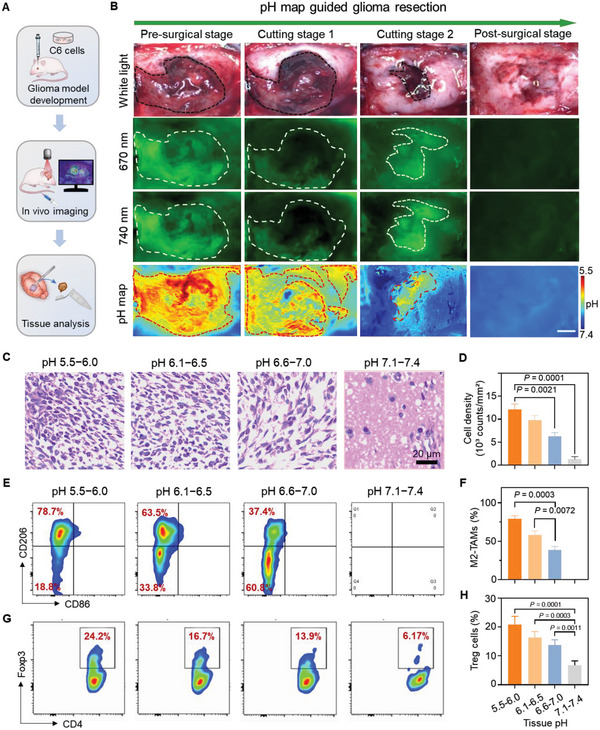
CP2‐M guided surgery by delineating pH map of tumor bed. A) The procedure of CP2‐M guided surgery in rat models bearing C6 glioma allografts. B) Intracranial surgery with the guidance of a pH map delineated by CP2‐M. The surgery did not terminate until all the regions with pH values below 7.1 were excised, scale bars: 2.0 mm. Representative C) H&E staining and D) cell density of the excised tissues with different measured acidities. Scale bar: 20 µm. Flow cytometric analysis of immunostimulatory E,F) CD206+ CD86‐ macrophages (M2‐TAMs) among CD11b+ F4/80+ cells and G,H) Foxp3+ CD4+ Tregs among CD45+ CD3+ cells in the excised tumor tissues with different acidities. Data are shown as the mean ± s.d. (*n* = 3), and an unpaired t‐test is used for comparison between two sets of data in D, F, and H. Statistical significance was considered for a *p*‐value of < 0.05.

The image‐guided surgery was performed in C6 GBM allograft bearing rat models post administration of fluorescence probe. After the image‐guided surgery, tumor tissues were excised following the flow cytometry and immunohistochemical staining analysis (**Figure** **7**A). The tumor‐bearing rat models were randomly divided into three groups for surgical resection under the guidance of ICG, 5‐ALA, and CP2‐M, respectively (Figure 7B, Figure S15). Figure 7B demonstrated the representative white light, fluorescent images, and pH maps of the exposed GBM allograft at different stages during the CP2‐M‐guided surgery. The surgical resection didn't stop until all the regions with pH values below 7.0 were excised. The excised tissue specimens were divided into 4 groups according to their acidities and examined by histological analysis (Figure 7C). High densities of tumor cells were observed in the acidic tissues (pH 5.5–6.5), but they were hardly found in tissues with pH above 7.1 (Figure 7D). Flow cytometry studies demonstrated the tissue acidity correlated M2‐TAMs density (Figure 7E). The percentage of immunosuppressive CD206^+^CD86^−^ TAMs was 78.7% in the tissues with pH 5.5–6.0, but this value decreased to 37.4% in tissue with pH 6.5–7.0. In contrast, the CD206^+^ CD86^−^ TAMs were barely detected in neutral tissues (pH 7.1–7.4) (Figure 7F). Notably, tissue acidity‐dependent Treg cell proportions in total T cells were also evident (Figure 7G). This value increased 4‐fold from 6.2% in tissue with pH 7.0–7.4 to 24.2% in tissue with pH 5.5–6.0 (Figure 7H). The above studies imply the feasibility to define glioma infiltrative margins via CP2‐M generated pH map.

### 2.7 CP2‐M Guided Surgery Inhibits Glioma Recurrence

**Figure 8:**
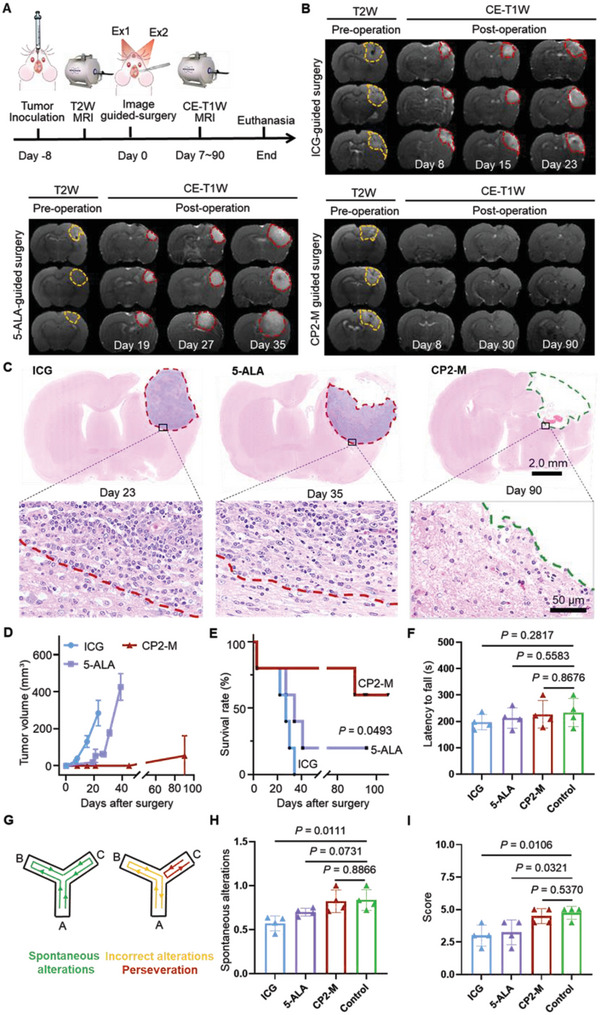
CP2‐M guided surgery suppresses tumor recurrence. A) Experimental timeline for monitoring tumor relapse after image‐guided surgery in rat models bearing intracranial GBM allograft. B) Representative T2W and contrast‐enhanced T1W MR images of rat models at selected days post‐ICG, 5‐ALA, or CP2‐M guided surgery. Tumor margins are indicated by dashed line. The mass at the tumor bed after CP2‐M guided surgery was verified as hydrocephalus instead of relapsed tissue. C) Representative H&E staining images of rat brain sections at the end of the experiments. The red dotted line indicates tumor recurrence after surgery, while the green dotted line represents the tumor excision bed that did not recur. The scale bars are 2.0 mm and 50 µm (enlarged). Relapsed tumor volume D) and Kaplan–Meier survival curves E) of rat models after surgical intervention (*n* = 5 rat models per group). F) Time spent on the rotarod by mouse models after surgery guided by ICG, 5‐ALA, or CP2‐M. G) Diagrams for the spontaneous (left) and Incorrect (right) alternation in the Y‐maze test. H) The spontaneous alternation performance of mouse models in the Y‐maze test. The percent of alternation in the Y‐maze was significantly above chance level (50%) in CP2‐M group but not in ICG and 5‐ALA group. I) Beam walking scores in the beam‐walking test after surgery (*n* = 4 mice per group). CE‐T1W: contrast‐enhanced T1‐weighted; MRI: magnetic resonance imaging. Data are given as the mean ± SD (*n* = 4 or 5 mice per group), the statistical significance of the survival curve was calculated using the log‐rank test, and two‐tailed Student's t‐test was performed in D, F, H, and I. Statistical significance was considered for a *p*‐value of <0.05.

Tumor volumes before and after the image‐guided surgery were monitored by the contrast‐enhanced T1W MRI (**Figure** **8**A). The prognosis of the ICG group was abysmal with rapid tumor relapse for all animals in one week after surgery (Figure 8B). The 5‐ALA group showed a better prognosis than the ICG group, with varying degrees of tumor recurrence at three weeks post‐surgery. In contrast, tumor recurrence was only observed in one of the tested animals in the CP2‐M group. At the end of the experiment, H&E staining of rat brain sections verified the MRI manifestations (Figure 8C). No residue cancer cells were detected in the tumor cutting edge even 90 days post‐CP2‐M guided operation. Figure 8D showed the volume of the recurrent tumor as a function of time post‐operation. The average tumor volume of the ICG group increased from 32.8 mm^3^ on day 8 to 130.9 mm^3^ on day 15 post‐surgery. Meanwhile, the relapsing tumor volume of the 5‐ALA group increased from 27.7 mm^3^ on day 19 to 534.6 mm^3^ on day 35. Significantly, no tumor recurrence was found in the CP2‐M group within 1 month after surgery, and the recurrence rate was 20% until day 90. The Kaplan–Meier survival curve showed that while the animals in the ICG group exhibited a median overall survival (OS) of 27.0 days, the animals in the 5‐ALA group had a median OS of 34 days. Remarkably, 60% of animals in the CP2‐M group survived above 90 days (Figure 8E) and no tumor recurrence was observed by MRI. The rats in all three groups showed a steady weight gain until the recurrence happened (Figure S16, Supporting Information). Histological H&E staining did not demonstrate any pathological damage to rat major organs at 14 days post injection of CP2‐M with a dose of 5 µmol per kg body weight (Figure S17, Supporting Information). These studies indicate the tolerable acute toxicity of CP2‐M.

To investigate the potential effect of surgery on motor dysfunction and memory, a series of tests including the rotarod test, Y‐maze test, and Beam walking were conducted on both control rats and rat models following the surgical procedure. In CP2‐M‐guided surgery group, no obvious motor deficits were observed since they performed well in the rotarod test (Figure 8F). To assess the spatial memory, the percentage of triads of arm entries in which the mouse sequentially visited each possible arm without repeating was recorded (Figure 8G). For spontaneous alternation, both control and CP2‐M‐guided surgery rats showed a percentage of alternation significantly higher than chance level, whereas this was not observed in other groups of mice (Figure 8H). Furthermore, both the rats in the CP2‐M group and the control group could pass the balance beam smoothly and performed better in the Beam walking score than other groups (Figure 8I). These findings indicate that CP2‐M‐guided surgery resulted in tolerable neurological damage to the brain. Overall, CP2‐M guided surgery might achieve more completed tumor resection than the clinically used imaging strategies, thereby preventing glioma recurrence and improving surgical prognosis.

## 3 Discussion

The accurate location of infiltrative tumor margins is vital for enhancing the surgical prognosis of glioma. Current strategies rely on indicators such as cancer cell‐associated receptors, enzymatic activity, metabolic products, and vascular disruption to define glioma margins. For example, MRI‐based neuro‐navigation and ICG‐based fluorescence imaging detect tumors through the extravasation of imaging probes from the disrupted brain vasculatures. However, the spatial mismatch between the regions of BBB disruption and tumor infiltration often leads to incomplete excision and rapid recurrence. Probes targeting specific receptors of glioma, such as epidermal growth factor receptor VIII (EGFR VIII), have been developed to demarcate tumor margins.^[^ ^27^ ^]^ Nevertheless, the heterogeneous distribution of the receptors within tumors compromises imaging accuracy. To address this issue, our previous works proposed a novel “metabolic margin” guided surgery strategy, which visualizes glioma invasive boundaries by sensing tumor‐associated metabolites. We developed a series of pH‐responsive surface‐enhanced Raman scattering (SERS) probes to define glioma margins by sensing the acidic extracellular fluid.^[^ ^28^, ^29^, ^30^ ^]^ In addition, 5‐ALA has been approved for clinical use in guiding glioma surgery by utilizing upregulated enzymatic activities in cancer cells to convert it to fluorescent protoporphyrin. While these strategies show promise in improving surgical prognosis, they face a common challenge of false negatives due to reduced cancer cell density in the infiltrative tumor region. This work puts forward a new strategy to identify glioma infiltrative margins by minimizing M2‐TAMs instead of cancer cells, thereby minimizing the false negatives.

TAMs represent the most abundant innate immune population in glioma and actively contribute to cancer cell infiltration. First, TAMs are actively involved in the regulation of epithelial‐mesenchymal transition (EMT), an event presenting the initiation of tumor invasion with characteristics including the loss of junctions between cancer cells, attenuation of apical‐basal polarity, and acquiring a motile mesenchymal cell phenotype.^[^ ^31^ ^]^ Studies have shown that TAMs tend to infiltrate abundantly at EMT hotspots, such as the edge of tumor nests.^[^ ^32^ ^]^ Second, TAMs facilitate the migration of cancer cells by degrading the extracellular matrix (ECM) and compromising Cell‐ECM interactions through the secretion of numerous proteolytic enzymes including matrix metalloproteinase*s* (MMPs), cathepsins, and serine proteases.^[^ ^32^, ^33^ ^]^ Third, TAMs are the key cells that create an immunosuppressive tumor microenvironment and protect the cancer cells from the cytotoxic effect of the T cells. Given the pivotal role of M2‐TAMs in facilitating cancer cell invasion and their enrichment at glioma invasive margin, targeting TAMs holds great promise for improving surgical prognosis.

TAMs can be categorized into tumor‐promoting and tumor‐suppressive phenotypes. M1‐TAMs are involved in pathogen defense through the secretion of reactive oxygen species and pro‐inflammatory cytokines, whereas M2‐TAMs facilitate tissue repair and angiogenesis by producing growth factors and anti‐inflammatory cytokines.^[^ ^34^ ^]^ The majority of TAMs belong to M2‐TAMs that actively maintain a suppressive tumor immune microenvironment (TIME) that negatively impacts current immunotherapeutic strategies. Notably, macrophage polarization is accompanied by metabolic reprogramming, wherein cells adapt their metabolic pathways to support their energy and material needs.^[^ ^35^ ^]^ This phenotype‐dependent glucose metabolism results in the distinct acidification of the phagosomes in the macrophages.^[^ ^36^ ^]^ M1‐TAMs prefer glycolysis and pentose phosphate pathway, which activates NOX2, a phagosomal membrane‐bound enzyme complex. NOX2 catalyzes the production of ROS by transferring electrons from NADPH to oxygen. The dismutation of ROS into hydrogen peroxide consumes a large number of protons, resulting in the neutralization (pH in the 7.2‒7.5 range) of the phagosome lumen. In contrast, M2‐TAMs are more dependent on oxidative phosphorylation, which leads to an acidic phagosomal environment (pH in the 4.5‒5.5 range).^[^ ^22^ ^]^ Given the phenotype‐dependent phagosomal acidity, it is feasible to differentiate M2‐TAMs from M1‐TAMs and surrounding cancer cells by monitoring their phagosomal pH values.

In comparison to ICG and 5‐ALA, CP2‐M visualizes glioma infiltrative margins with a higher T/B ratio and prolonged time window. The performance of CP2‐M can be explained by the below reasons. First, the incorporation of the PEG linker optimizes the pharmacokinetics of the imaging probe. Locating the tumor infiltrative region within an extended time window helps to excise the tumor foci accurately and completely. Second, mannose exhibits excellent biocompatibility and high binding affinity to CD206, a specific biomarker of M2‐TAMs. Third, an increased T/B ratio was obtained with a hierarchical targeting strategy.^[^ ^37^ ^]^ This strategy consists of three stages including (1) passive accumulation in tumor tissue via enhanced permeability and retention (EPR) effect; (2) targeting M2‐TAMs by binding to their membrane receptor CD206; (3) fluorescence activation in the acidic phagosomes of M2‐TAMs after internalization. In this way, CP2‐M holds the promise to overcome the false negatives of the infiltrative region.

## 4 Conclusion

In this work, we reveal the accumulation of M2‐TAMs in glioma‐invasive margins and propose a hierarchical strategy to intra‐operatively define glioma margins by visualizing M2‐TAMs instead of cancer cells. To achieve above goal, we develop a pH ratiometric fluorescence probe that specifically identifies M2‐TAMs by sensing their acidic phagosomal lumen. The utilization of this probe demonstrates improved surgical prognosis in comparison to currently used imaging probes in clinical settings. Our hierarchical strategy has the potential to revolutionize the paradigm of tumor‐targeting imaging, addressing the existing challenges in imaging infiltrative glioma margins characterized by sparse distribution of cancer cells. This approach holds promise for precise image‐guided surgery, aiming to reduce postoperative recurrence rates and prolong patient survival.

## 5 Experimental Section

### Synthesis and Characterization of Imaging Probes

See the Supplementary Information for the detailed synthesis characterization information.

### Glioma Datasets and Preprocessing

Gene expression profiles of specimens with clinical information were downloaded from The Cancer Genome Atlas (TCGA; http://cancergenome.nih.gov/), Repository for Molecular Brain Neoplasia Data (Rembrandt; http://rembrandt.nci.nih.gov), and the Chinese Glioma Genome Atlas (CGGA; http://www.cgga.org.cn) databases for further analyses. Moreover, the corresponding patient survival information, histology, and pathological grades were also obtained.

### Analysis of Immune Cells in Glioma

Fraction Cell‐type Identification By Estimating Relative Subsets Of RNA Transcripts (CIBERSORT) web tool (https://cibersort.stanford.edu/), a computational method that accurately resolves the abundance of diverse cell subsets, was applied to calculate the relative fractions of 22 types of tumor‐infiltrating immune cells with 1000 permutations by using the CIBERSORT package in R language.^[^ ^38^ ^]^ In this study, CIBERSORT with 1000 permutations was applied to explore the density of 22 immune cells based on the three datasets from the public databases, and the fraction of 22 immune cells between LGG and GBM index groups was performed using Student's t‐test. *p* < 0.05 indicated a significant difference.

### Collection of Clinical Specimens

The experiment on glioma tissue samples from patients was carried out in accordance with the ethical regulations of Huashan Hospital Affiliated with Fudan University (No. 256, 2015). The pathological diagnosis was based on the 2021 World Health Organization (WHO) classification of glioma, in which glioma grade IV was considered GBM. All specimens were from patients who signed informed consent after surgery to approve the use of their tissues for research purposes. Human glioma tissue was considered exempt by the Human Investigation Ethics Committee of Huashan Hospital, Fudan University (No. 256, 2015).

### Histopathology and Immunohistochemistry Studies

For histopathological examination, the tissues (tumor, heart, liver, spleen, lung, and kidney) were fixed in 10% normal fixative formalin solution, dehydrated by series of alcohol (30–100%) and xylene for 1.5 h, and embedded in wax at 60 °C. The sections were then stained with hematoxylin and eosin (H&E). To observe the immune infiltration and anti‐inflammatory levels of tumor tissues, CD68 and CD206 immunohistochemistry (IHC) was performed according to the manufacturer's specifications (Yeasen Biotechnology (shanghai) Co.,Ltd., China). The positive cells were stained in brownish yellow and nuclei were counterstained in blue with hematoxylin. The sections were scanned by the VS200 digital slide scanner (Olympus, Japan) for observation of the staining results. The positive rate was quantified and analyzed with Image J software (NIH).

### Surface Plasmon Resonance (SPR) Studies

All SPR affinity assays were performed to investigate the binding kinetics of PEG_2K_‐mannose and CP2‐M to mannose receptor (CD206) protein with a Biacore T200 biomolecular interaction analyzer and CM4 sensor chip from GE Healthcare. The CM4 chips were activated with three sequential 1 min injections of 1.0 m NaCl/ 50 mM NaOH, and CD206 proteins were immobilized onto the flow cell of the CM4 chip by flowing it at 10 µL min^−1^ for 300 s in HBS‐EP+ running buffer (10 mM HEPES, 150 mM NaCl, 3 mM EDTA, and 0.05% (v/v) P20 surfactant, pH 7.4).^[^ ^39^ ^]^ The PEG_2K_‐mannose and CP2‐M were diluted in HBS‐EP+ buffer, filtered, degassed, and injected as a series of five two‐fold concentrations ranging from 0.78 µM to 12.5 µM. Kinetic experiments were performed at 25 °C with a flow rate of 30 µL min^−1^ in a running buffer. To regenerate the surface between two binding experiments, 10 mM glycine (pH 2.2) was injected for 30 s and washed with running buffer. The obtained sensorgrams were globally fitted over the whole range of injected concentrations, and response units at steady state were plotted against the corresponding analyte concentrations to generate the binding curve. Simultaneously *K*_d_ values were calculated by fitting the response versus concentration plots to a 1:1 steady‐state affinity model via the Biacore T200 analysis software.

### Cell Cultures

Murine glioma C6 cells, human glioma U251 cells, THP‐1 cells, and BV2 cells were purchased from the American Type Culture Collection (ATCC). Murine glioma C6 cells and human glioma U251 cells were cultured in a complete culture medium at 37 °C in a humidified atmosphere with 5% CO_2_. The complete culture medium refers to Dulbecco's modified Eagle's medium (DMEM, Hyclone) supplemented with 10% fetal bovine serum (FBS, Gibco), 1% penicillin‐streptomycin (P/S). THP‐1 cells in the monocyte state were differentiated into a macrophage‐like phenotype using THP‐1 complete culture medium with 100 ng mL ^−1^ phorbol‐12‐myristate‐13‐acetate (PMA) for 2 days. The THP‐1 complete culture medium refers to RPMI‐1640 (PM150110) supplemented with 10% FBS (Gibco), 0.05 mM β‐mercaptoethanol (PB180633) and 1% P/S. The PMA‐differentiated macrophages (M0) were primed with fresh medium supplemented with 40 ng mL ^−1^ IFN‐γ, 500 ng mL ^−1^ LPS for 1 day to differentiate into M1 phenotype, or with 40 ng mL ^−1^ IL‐4 for 2 days into the M2 phenotype. The BV2 cells were treated with IL‐4 (40 ng mL ^−1^) for 2 days to polarize into M2 phenotype or treated with LPS (400 ng mL ^−1^) and IFN‐γ (40 ng mL ^−1^) for 1 day to polarize into M1 phenotype.

### Isolation and Polarization of Bone Marrow‐Derived Macrophages (BMDM)

Femur and tibia bones were obtained from C57 mice aged 6 to 8 weeks. The end of the bones was then surgically dissected, and the bone marrow was mechanically flushed out repeatedly with a 1.0 mL syringe with a 22 G needle. It was then filtered through a 70 µm cell strainer and centrifuged at 2,000 rpm for 2 mins. Then cells were resuspended and cultured in a BMDM growth medium (complete culture medium with 10% FBS, 1% penicillin‐streptomycin, and 10 ng mL ^−1^ M‐CSF) at 37 °C, 5% CO_2_. Seven days later, BMDMs were treated with IL‐4 (40 ng mL ^−1^) to stimulate M2 polarization for 2 days, or treated with LPS (400 ng mL ^−1^) and IFN‐γ (40 ng mL ^−1^) to stimulate M1 polarization for 1 day.

### Cytotoxicity Assay

Cell Counting Kit (CCK)−8 assays (Dalian Meilun Biotechnology) was used to assess cell viability. Briefly, a culture medium containing probe CP2‐M with concentrations of 6.25 to 50 µM was added to cells seeded into a 96‐well plate and then incubated for 24 h. Then, 10 µL of CCK‐8 reagent was added to each well. The absorbance at 450 nm was recorded after co‐incubation for 4 h.

### Animal Experiments

All animal studies were conducted under the guidelines set by the Chinese Committee of Management of Laboratory Animals, and the overall project protocols were approved by the Ethics Committee of Fudan University School of Pharmacy (2019‐03‐FY‐LC‐01).

### Pharmacodynamics/Pharmacokinetics (PK/PD) Analysis

For the in vivo PK study of the probes, 300 µL of CP2 or CP2‐P or CB‐M, or CP2‐M was intravenously injected into healthy rats (male, 180–200 g) with a dose of 5.0 µmol kg^−1^, respectively. Subsequently, 300 µL blood was collected from every rat at 1, 5, 10, 15, 30 min, 1, 2, 4, 8, and 12 h after administration. Then the blood samples were centrifuged at 1500×g for 10 min, and the probe was extracted from the serum using acetonitrile. Finally, the integrated fluorescence intensity of the probes was measured via a fluorescence spectrophotometer. The concentration‐time profile of each probe was plotted and the area under the curve up to the last measurable concentration (AUC_0‐tlast_) was calculated using the linear trapezoid method.

The population pharmacodynamics/ pharmacokinetics (PK/PD) model was developed by the nonlinear mixed‐effects modeling software (NONMEM, version 7.5, ICON Development Solutions, MD, USA) using the first‐order conditional estimation with interaction (FOCE‐I) estimation method.^[^ ^41^ ^]^ Perl‐speaks‐NONMEM (PsN, version 5.2.6, Uppsala University, Sweden) and Pirana (version 3.0.0, Certara, USA) were used to facilitate the use of NONMEM. For statistical analysis and output visualization, R (version 4.1.1, R Foundation for Statistical Computing, Vienna, Austria) and the R packages tidyvpc (version 1.2.0, Certara, USA) were employed. Due to the profound influence of PEG_2K_ modification on the probe's PK behavior, the population PK model was developed for PEG_2K_‐modified probes (CP2‐M, CB‐M, and CP2‐P) and PEG_2K_‐free probe (CP2), respectively. One‐, two‐ and three‐compartment PK models were tested. The model was selected based on the AIC, objective function value (OFV), GOF plots, and visual predictive check (VPC). Furthermore, the effect of mannose modification on the PK behavior of the probes was assessed by the stepwise covariate modeling (SCM) tool in PsN.^[^ ^42^ ^]^ Significance levels of 0.05 (ΔOFV>3.84) and 0.01 (ΔOFV>6.64) were used for the forward selection and the backward elimination process, respectively.

The observed delay between the probe concentration (PK) and the ratio of fluorescence signal to background noise (T/B ratio, PD) was described by the biophase model via the incorporation of a hypothetical drug distribution process from plasma to the target site.^[^ ^43^ ^]^ The ratio of target and plasma concentration was then linked to the T/B ratio. Due to the high permeability of the small molecule probe CP2, the equilibrium condition of the biophase model was derived to describe the steady T/B ratio from CP2. On the other hand, the kinetic biophase model was adopted for the remaining three PEG_2K_ modified probes CP2‐M, CM‐B, and CP2‐P. Similar to the PK model development, the final PK/PD model was selected based on AIC, GOF plots, VPC, and/or biological knowledge and interpretation. Furthermore, the simulation of the final PK/PD model was performed to explore the optimal time window for imaging within 10 h after a single injection of 3 mg CP2‐M. The detailed model description and NONMEM code were provided in the supplementary material.

### Development of Animal Models Bearing Orthotopic Glioma Allograft/Xenograft

The orthotopic glioma models were initially established by implanting murine glioblastoma C6 cells into rats or human glioblastoma U251 cells into nude mice intracranially with a skull‐puncture technique. First, the rat glioblastoma C6 cells or human glioblastoma U251 cells were kept in a 37 °C humidified incubator (Thermo, USA) with 5% CO_2_. Male Sprague Dawley rats (SD rats,160−180 g) and nude mice (18−22 g) were purchased from Shanghai Slac Lab Animal Ltd (Shanghai, China) and kept under specific pathogen‐free (SPF) conditions with ready access to standardized food and water.^[^ ^44^ ^]^ After inhalation anesthesia with isoflurane in SD rats, 5 µL of C6 glioblastoma cells (a total of 5 × 10^5^ cells suspended in 30 µL PBS) were stereo‐tactically injected into the right cerebral hemisphere at 4.0 mm from the lateral side of the bregma and 4.0 mm depth from the brain surfaces of the rat by using a 10 µL Hamilton syringe. To establish a xenograft model of human glioma in nude mice, 5 × 10^5^ cells human U251 cells suspended in 40 µL PBS were inoculated into the right cerebral hemisphere at 3 mm from the lateral side of the bregma and 3.5 mm depth from the brain surfaces of BALB/c nude mice. The injection speed was 3.0 µL min^−1^, and the syringe was retained at the injection site for 5 min to avoid backflow after injection. At ≈7–10 days post‐inoculation, orthotopic glioma model establishment was confirmed by T2W‐MRI.

### In Vivo/Ex Vivo Fluorescence Imaging Studies

Three hours after the intravenous injection of probe via the tail vein, tumor‐bearing rat models were sacrificed to evaluate the efficacy of the probe in tracing glioma infiltrating boundaries. To obtain fluorescence images of the brain, we exposed the brains of the rat models to the laser and then irradiated them with excitation light of 740 and 670 nm respectively. The ratio of two fluorescence images was converted to a pH map pixel by pixel. Whole body fluorescence images of U251 glioma‐bear nude mouse models were collected using an In Vivo Imaging System (Xenogen, USA) (Ex/Em: 740 nm/800 nm) at 0.5, 1, 2, 4, 8 h post intravenous injection of probe with a fluorophore counted dose of 5.0 µmol kg^−1^. For the tissue distribution study, the mice were sacrificed, and brain tumors and major organs were collected for ex vivo imaging.

### Flow Cytometry Analysis of Tumor Tissues

Glioblastoma tissues were excised followed by homogenized by mechanical grinding and passing through a 70‐µm cell strainer to obtain single‐cell suspensions. Cell suspensions were centrifuged at 3000 rpm for 3 min at 4 °C, washed with PBS, blocked with 5% bovine serum albumin (BSA) in PBS, incubated with corresponding antibodies for 20 min, and washed 3 times. Total TAMs were defined as CD11b+ F4/80+ in CD45+ leukocytes from tumor tissues. The M1 polarized TAMs were gated as CD206‐CD86+ in total TAMs, while the M2 polarized TAMs were gated as CD206+CD86‐ in total TAMs. All data were collected on a flow cytometer (BD, FACS Aria II) and analyzed with FlowJo V10 software (BD Biosciences, USA).

### Confocal Microscope Imaging Studies

C6 and U251 cancer cells were seeded onto glass‐bottom Petri dishes and incubated for 24 h at 37 °C under 5% CO_2_. BMDMs were incubated for 7 days and treated with IL‐4 for 2 days as previously mentioned.^[^ ^40^ ^]^ BV2 cells and THP‐1 cells were polarized as described above. Different concentrations of probes were added at different times. All images were from Laser Scanning Confocal Microscope (Carl Zeiss LSM710). A 63 X oil immersion objective lens was used. CP2‐M was excited by a 647 nm laser, and DAPI was excited by a 405 nm laser.

### In Vivo Fluorescent Image‐Guided Surgery

The orthotopic C6 glioma tumor‐bearing rats were randomly assigned to three groups for fluorescence imaging experiments: 5‐ALA group, ICG group, and CP2‐M group (*n*  =  3 SD rats for each probe). After the tumor‐bearing rats were anesthetized with isoflurane at 2% concentration‐delivered with medical air through a vaporizer, and then craniotomy was performed for fluorescent imaging and surgical removal of the brain tumor. ICG and CP2‐M (600 µL, 5 µmol kg^−1^) were injected into the tumor‐bearing rats via the tail vein. 5‐ALA (20 mg kg^−1^, 30 mg 5‐AlA·HCl mL ^−1^) was orally administered as recommended by the FDA.^[^ ^45^ ^]^ In vivo Fluorescence imaging was performed with a macro view fluorescent stereomicroscope (MVX10, Olympus, Japan) equipped with a camera (BIOHD‐EPS, Fluoca, Singapore), which was controlled by the ImspectorPro Software (LaVision BioTec) and the intelligent automatic processing program. Time‐dependent in vivo fluorescence images in the brains of tumor‐bearing rats were captured at 0.5, 1, 2, 4, and 6 h post‐treatment. In the 5‐ALA and ICG groups, tumor boundaries were differentiated for resection according to fluorescence intensity. In the CP2‐M group, the fluorescence intensity ratio excited at 740 and 670 nm was converted into a pH distribution map by a built‐in calibration curve. Under the guidance of the pH map, all tissues with a pH below 7.0 were removed according to pH mapping.

The specific acquisition parameters are as follows: full wavelength laser source (PE4000, Cooled, England), two excitation filters central wavelength at 670 and 740 nm, respectively; emission filter 790 nm (bandwidth 70 nm), exposure time 1.0 s. The central wavelength of the ICG excitation filter is 710 nm (bandwidth 75 nm), and the wavelength range of the emission filter is 765–855 nm.^[^ ^46^ ^]^ 5‐ALA excitation was performed at 385 nm (range 360–410 nm), not at the reported optimal 405 nm wavelength, and emission wavelength ranged at 595–665 nm.^[^ ^47^ ^]^ Tumor‐bearing rats were euthanized at the end of the study. The heart, liver, spleen, lung, kidney, and brain were removed, and fluorescence imaging was performed in vitro for biodistribution.

### Ex Vivo Fluorescence Imaging and Microscopy Studies

The removed brain was then rapidly immobilized in 4% paraformaldehyde in PBS, embedded in paraffin, sectioned, and stained with H&E according to standard protocols. The H&E‐stained brain section images were taken by an optical microscope (Olympus), which were further compared with a pH map to determine whether the glioma infiltrating boundary defined by CP2‐M matches the actual boundary indicated by the pathological result. The removed brain was made into frozen sections, and the CP2‐M signal and bright‐field images were taken by confocal microscopy to verify probe entry into the brain. The altered frozen sections were subjected to immunofluorescence staining to verify the co‐localization of CP2‐M with CD206 in vivo, antibody information: CD206 (proteintech, 60143‐1, 1:50).

### MRI Studies

MRI studies were performed at day 10 post‐cancer cell inoculation and on postoperative days 8, 15, 22, 30, and 90 using an 11.7 T BioSpec 117/16 USR MRI system (Bruker, Ettlingen, Germany). The rats were anesthetized with 2%‒3% isoflurane in 20% oxygen, and the respiration was monitored and maintained at 60‒90 min^−1^ during the MRI scanning. Successful tumor inoculation was verified by T2‐weighted images, and contrast‐enhanced T1‐weighted imaging with gadolinium agent was used to monitor tumor recurrence. The tumor outline in every slice was drawn as the region of interest (ROI) by experts using a free open‐source software package (ITK‐SNAP, version 3.6.0; http://itksnap.org) for computer‐based image analysis. Tumor volume was calculated by multiplying the number of voxels within each ROI by the voxel size (0.003 mm^3^) in outlined slices, and euthanasia was performed when the tumor volume grew >500 mm^3^. All of the code developed for processing was written in Python (version 3.8.11).

### Behavioral Testing

Behavioral tests were performed on the post‐operative mice in the following order: beam‐walking test (BWT), Y‐maze test, and rotarod test. The effect of surgery on body balance and coordination was examined 10 days post‐surgery using the BWT. A balance beam, measuring 1.0 meters in length and 14 millimeters in width, was used. The time taken by the mice across the balance beam and number of slips they made within 60 s were recorded. The tested mice were scored according to the most commonly used Feeney's scale (1–5) to assess their performance on the test. A spontaneous alternation Y‐maze test was conducted to assess the spatial memory of tested mice. Mice were placed at the end of one arm of a symmetric Y‐maze and given 5 min to freely explore the three arms of the Y‐maze. The percentage of alternations were determined by dividing the number of successive entries into the three arms of the Y‐maze by the total number of entries. The rotarod test was employed to assess motor dysfunction by measuring the time to fall from the rotarod system (XR1514, Xinruan, Shanghai, China). The rotarod test was carried out using an accelerating protocol from 4 to 50 rpm over the first two min of the test and then remained at the maximum speed for the remaining three min. The mice were placed on the rotarod, and the time it took for them to fall off the apparatus was recorded as the latency to fall. All behavioral testing was conducted during the light phase. Prior to behavioral testing, mice were acclimated to the procedure room for at least 2 h. The behavior of the mice was recorded and stored for offline analysis using Ethovision XT 12 software (Noldus Information Technology, Wageningen, The Netherlands).

### Statistical Analysis

All statistical analyses were performed with GraphPad Prism software version 8.0 through the unpaired student's t‐test, one‐way or two‐way ANOVA with post hoc multiple comparisons. The statistical significance of the survival curve was calculated using the log‐rank test. For all statistical tests, statistical significance was indicated as NS  =  non‐significant, *p< 0.05, **p< 0.01, ***p< 0.001, and ****p< 0.0001. Statistical significance was considered for a *p*‐value of <0.05.

## Conflict of Interest

The authors declare no conflict of interest.

## Supporting information

Supporting Information

## Data Availability

The data that support the findings of this study are available from the corresponding author upon reasonable request.
