# Correction to “Intra‐Operative Definition of Glioma Infiltrative Margins by Visualizing Immunosuppressive Tumor‐Associated Macrophages”

**DOI:** 10.1002/advs.202500244

**Published:** 2025-04-25

C. Cao, H. Yin, B. Yang, Q. Yue, G. Wu, M. Gu, Y. Zhang, Y. Fan, X. Dong, T. Wang, C. Wang, X. Zhu, Y. Mao, X.‐Y. Zhang, Z. Lei, C. Li. Intra‐Operative Definition of Glioma Infiltrative Margins by Visualizing Immunosuppressive Tumor‐Associated Macrophages. *Adv. Sci*. **2023**, 10, 2304020.

https://doi.org/10.1002/advs.202304020

Following the publication of this article, we conducted a comprehensive review and archiving of the raw data, during which were regrettably identified errors in Figures 4 and 7 and Figure S5 (Supporting Information). These errors were unintentionally introduced while organizing the figures, and the corrected versions of these figures are provided as follows:

In Figure 4C, the “CP2‐P (1 h)” image was inadvertently duplicated from the “CP2 (0.5 h)” during the assembly of Figure 4C. The correct figures are shown below.


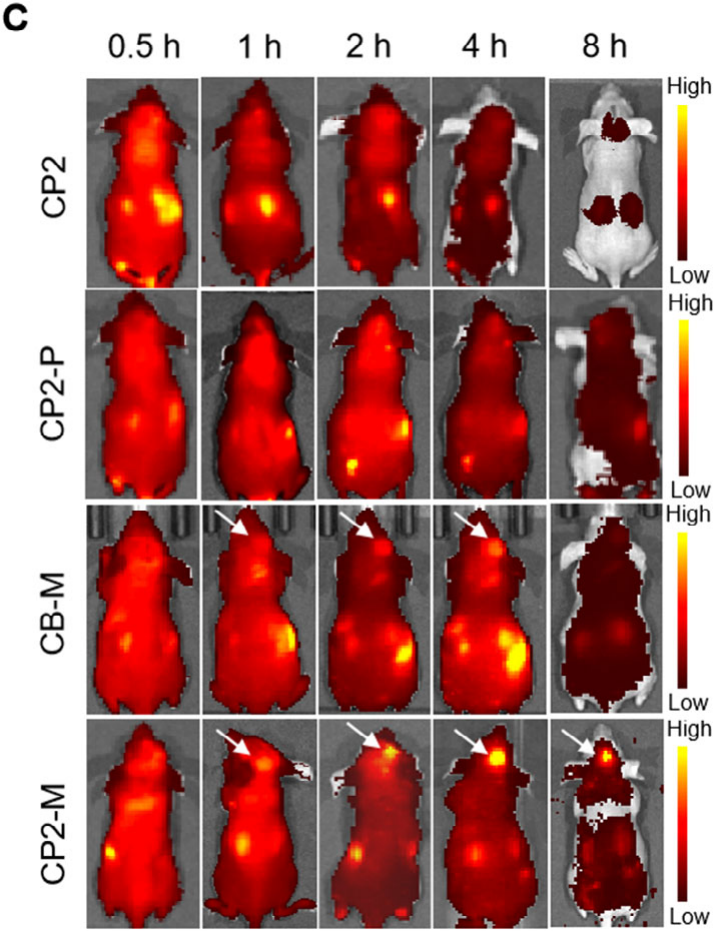


In Figure 7C, the “pH 5.5‒6.0” image was mistakenly chosen from the group “pH 6.1‐6.5” during the layout of the images. The corrected figures are shown below.


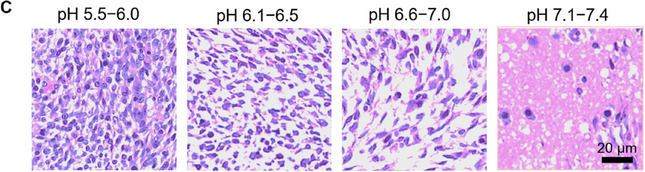


In Figure S5 (Supporting Information), the fluorescence image of CP2‐P (3 h) was inadvertently duplicated from the image of CP2‐M (3 h) during the layout of the images. The correct figures are shown below.


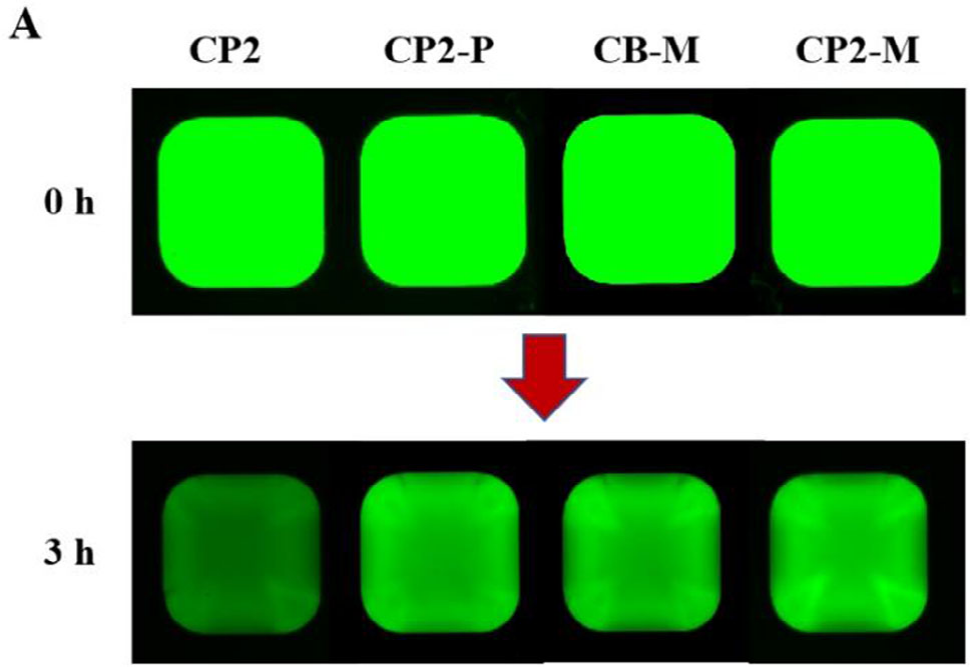


This correction does not affect the results or conclusions of the original article.

We apologize for this error.

## Supporting information



Supporting Information

