# Supplementary material for: Correction to “Intra‐Operative Definition of Glioma Infiltrative Margins by Visualizing Immunosuppressive Tumor‐Associated Macrophages”
Source: Adv Sci (Weinh). 2025 Apr 25;12(19):2500244. doi: 10.1002/advs.202500244 (PMC12097088; doi:10.1002/advs.202500244)
Supplement: Supplementary file 1 — Supporting Information [file ADVS-12-2500244-s001.pdf]

ADVANCED SCIENCE
Open Access

Supporting Information

for *Adv. Sci.*, DOI 10.1002/advs.202500244

Correction to “Intra-Operative Definition of Glioma Infiltrative Margins by Visualizing Immunosuppressive Tumor-Associated Macrophages”

Supporting Information

Intra-operative Definition of Glioma Invasive Margins by Visualizing Immunosuppressive Tumor Associated Macrophages

Chong Cao†[a], Hang Yin†[a], Biao Yang†[a], Qi Yue†[a], Guoqing Wu[b], Meng Gu[a], Yuwen Zhang[c], Yang Fan[a], Xiaoyan Dong[a], Ting Wang[a], Xiao Zhu[a], Ying Mao*[a], Xiao-Yong Zhang*[c], Zuhai Lei*[a], Cong Li*[a,d]*

[a]Key Laboratory of Smart Drug Delivery Ministry of Education | Innovative Center for New Drug Development of Immune Inflammatory Diseases, Ministry of Education | School of Pharmacy | Department of Neurosurgery, Huashan Hospital, Fudan University, Shanghai 201203, China. Email: congli@fudan.edu.cn; lei_zuhai@fudan.edu.cn; maoying@fudan.edu.cn; xiaozhu@fudan.edu.cn

[b]School of Information Science and Technology, Fudan University, Shanghai 200438, China. Email: guoqingwu@fudan.edu.cn

[c]Institute of Science and Technology for Brain-Inspired Intelligence | MOE Key Laboratory of Computational Neuroscience and Brain-Inspired Intelligence | MOE Frontiers Center for Brain Science, Fudan University,Shanghai 200433, China. Email: xiaoyong_zhang@fudan.edu.cn

[d]State Key Laboratory of Medical Neurobiology | Zhongshan Hospital, Fudan University, Shanghai, China

†These authors contributed equally to this work.

Materials and Instruments:

Unless otherwise indicated, all chemical reagents were purchased from Bide Pharmatech Co.,Ltd., and anhydrous solvents were purchased from commercially sources without further purification. Methoxy PEG Amine (M-PEG-NH_2, MW2000) was obtained from JenKem Technology Co., Ltd (Beijing, China). Mannose PEG Amine (NH_2-PEG-mannose, MW2000) was purchased from Xi 'an ruixi Biological Technology Co.,Ltd. Ultrapure (Up) water, produced by a MT system (18.2 MΩ cm, Shanghai Leading Water Treatment Equipment Co., Ltd., China), was used throughout all the experiments. TLC Silica gel 60 F_{254} Aluminium sheets were purchased from Merck, and silica gel (200-300 mesh) for column chromatography was purchased from Qingdao Haiyang Chemical Co., Ltd., China. GIBCO

fetal bovine serum (FBS), Dulbecco's Modified Eagle Medium (DMEM), Roswell Park Memorial Institute 1640 (RPMI 1640), trypsin, streptomycin and penicillin were purchased from Shanghai Ruiyuan Biomedical Technology Co., Ltd. Cell Counting Kit-8 (CCK-8) was purchased from Dalian Meilun Biotechnology Co.,Ltd. LysoTracker Green DND-26 and MitoTracker Green FM were purchased from Yeasen Biotechnology (Shanghai) Co., Ltd, China. BCA Protein Assay Kit, SDS-PAGE Gel Preparation Kit, QuickBlock™ Primary Antibody Dilution Buffer and QuickBlock™ Secondary Antibody Dilution Buffer for Western Blot were purchased from Absin Bioscience Inc., China. CD206 protein for surface plasmon resonance was purchased from Shanghai Universal Biotech Co., Ltd., China. Immunol Staining Fix Solution and Permeabilization Buffer with Triton X-100 were purchased from Dakewe Biotech Co., Ltd, China. QuickBlock™ Blocking Buffer for immunohistochemistry and antifade polyvinylpyrrolidone mounting medium were purchased from Beyotime Biotechnology Co.,Ltd, China.

All pH measurements were carried out with a MP220 pH meter (Mettler Toledo, Switzerland). High-resolution mass spectra (HRMS) was measured with a Q-TOF 2 (Micromass, USA) or AB 5600^{+} Q-TOF mass spectrometer (AB Sciex, USA). High performance liquid chromatography (HPLC) analysis was conducted on LC-20A HPLC system (Shimadzu, Japan) equipped with SPD-M20A Photodiode Array Detector (190-800 nm). ^{1}H NMR spectra was recorded on a 400 MHz (Varian, USA) NMR spectrometer, and ^{13}C NMR spectra was recorded on a 600 MHz (Bruker, USA) NMR spectrometer. Electrophoresis apparatus trophoresis (BIO-RAD, USA) were used in Western Blotting studies. Flow cytometric studies were analyzed on BD (USA) FACSAria II flow cytometer. Zeiss LSM 710 META confocal laser scanning microscope (Carl Zeiss), ZEN 2012 software and ImageJ software were used in Confocal fluorescence imaging studies. Absorption and emission spectra were collected by UV-2550 UV-vis spectrophotometer (Shimadzu, Japan) and RF-5301PC fluorescence spectrophotometer (Shimadzu, Japan), respectively. The location of skull puncture is based on a stereotaxic apparatus (68025, RWD, China) during the establishment of glioma-bearing rat models. White light images of rat models were obtained by the stereomicroscope (Fluoca SZ51, China). In vivo fluorescence imaging experiments were performed on MVX10 stereomicroscope (Olympus, Japan). In vivo MRI studies were conducted by using a 11.7 T small animal micro-MRI scanner (Bruker BioSpec, Germany).

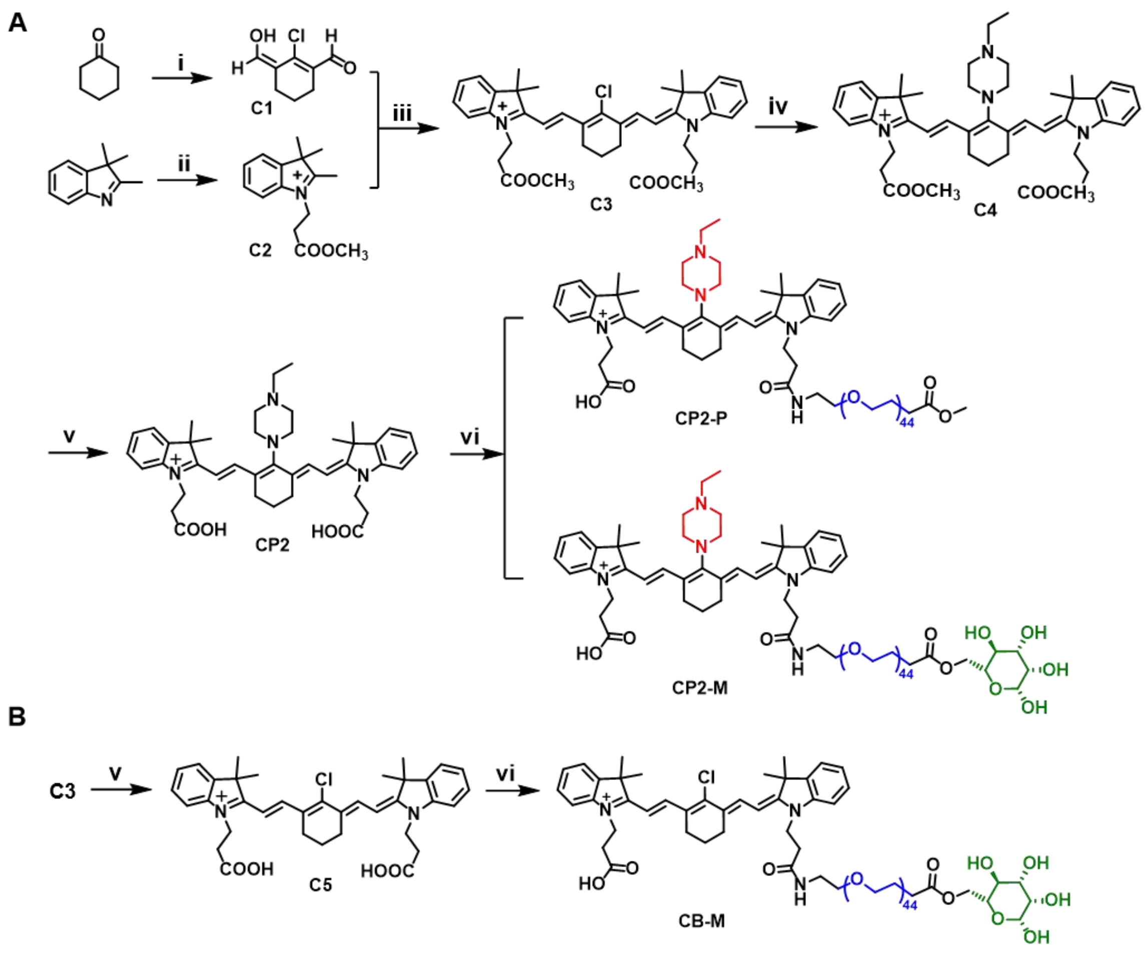

Scheme 1. Synthetic of CP2, CP2-P, CB-M, CP2-M. Reaction conditions: (**i**) DMF/$POCl_3$/DCM, 70 °C; (**ii**) methyl 3-bromopropanoate/ACN, refluxed overnight; (**iii**) NaOAc/acetic anhydride, 70 °C/1.0 h; (**iv**) N-ethylpiperazine, ACN, 60 °C/2.0 h; (**v**) NaOH/MeOH/H_2O, 0 °C /4 h; NH_4Cl/H_2O; (**vi**) DCC/NHS/NH_2-PEG_{2K}, r.t., (**vii**) DCC/NHS/NH_2-PEG_{2K}-mannose, r.t.. DMF: N, N-dimethylformamide; $POCl_3$: phosphorus oxychloride; DCM: dichloromethane; NaOAc: sodium acetate; NaOH: sodium hydroxide; NH_4Cl: ammonium chloride; DCC: dicyclohexylcarbodiimide; NHS: N,N-dimethylaminopyridine; r.t.: room temperature; ACN: acetonitrile.

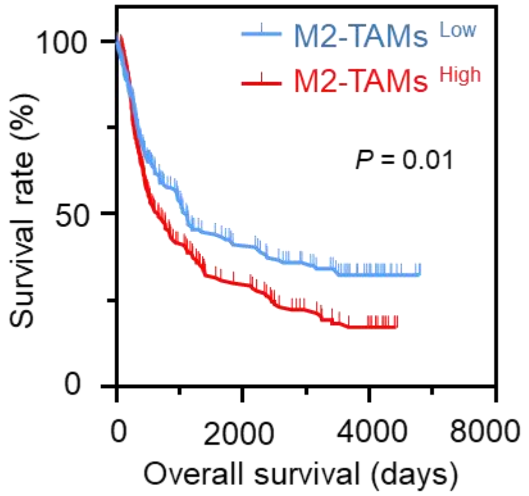

Figure S1. Survival curves of glioma patients as a function of TAM percentages in CGGA database.

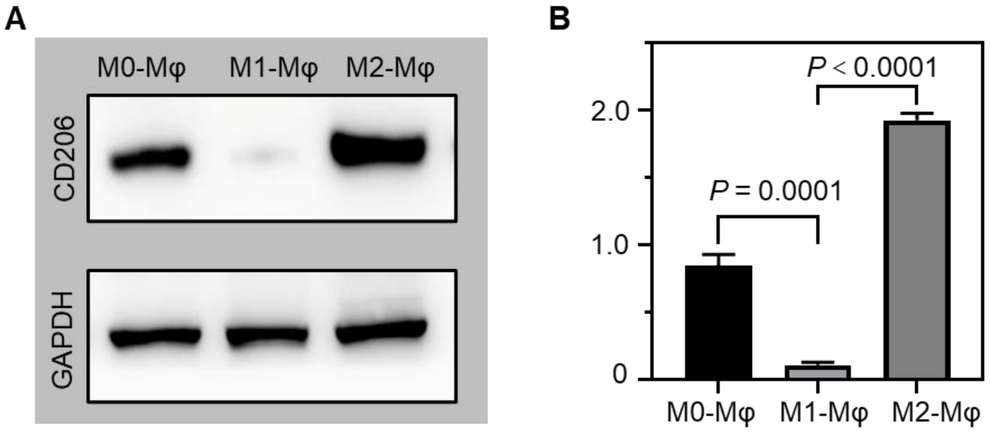

Figure S2. Representative western blotting analysis shows that CD206 is highly expressed in M2-Mφ compared to macrophages with other phenotypes.

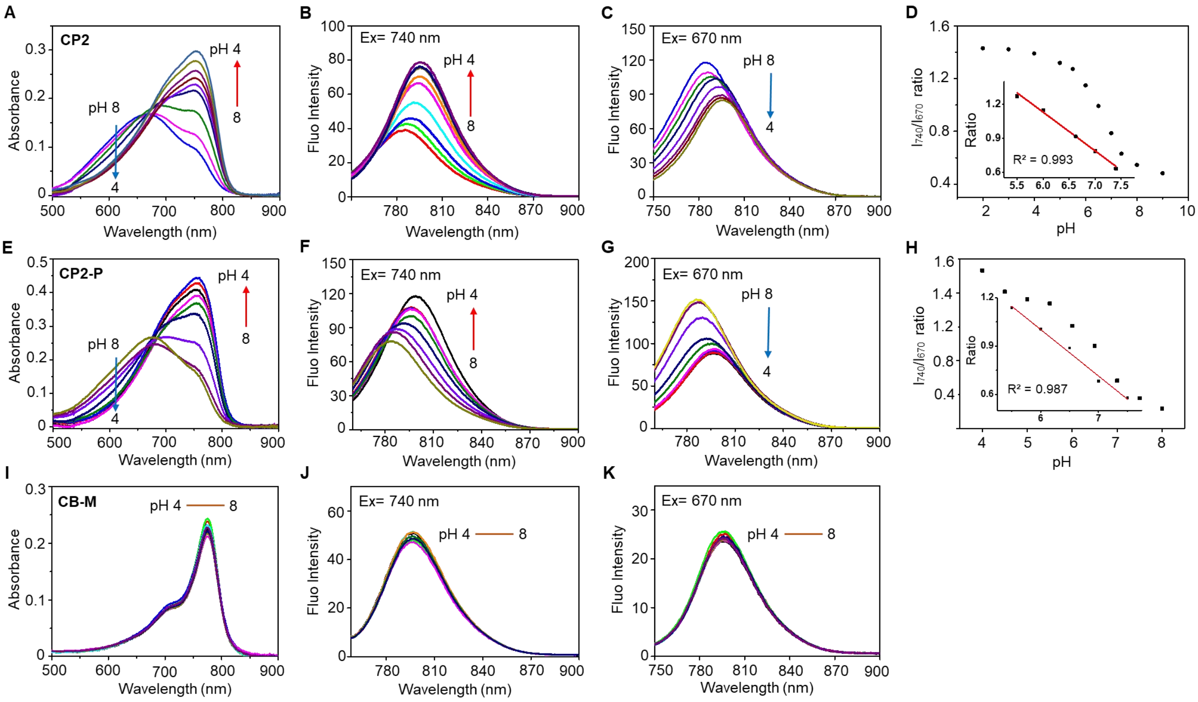

Figure S3. **The spectras of the control probe CP2, CP2-P, and CB-M.** Absorption (**A, E, I**) and fluorescence spectra (**B-C, F-G, J-K**) of CP2, CP2-P, and CB-M (2.0 μM) as a function of pH. CP2, CP2-P, and CB-M was excited at 740 nm and 670 nm, respectively. (**D, H**) Plotting fluorescence intensity ratio (I_{740}/I_{670} nm) of CP2 and CP2-P as a function of pH. Inset: the linear relationship between I_{740}/I_{670} ratio and pH value.

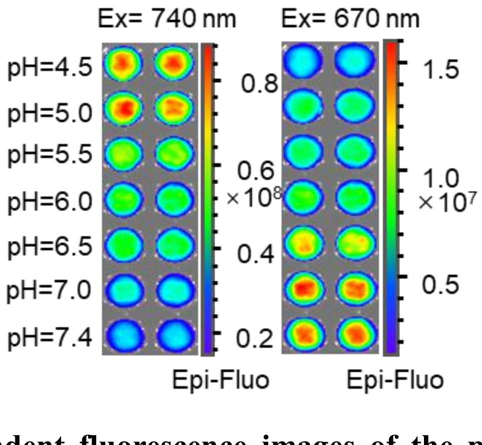

Figure S4. **The pH-dependent fluorescence images of the probe CP2-M.** Fluorescence imaging change of CP2-M (3.0 μM) after treated with different pH buffer solution. CP2-M was excited at 740 nm and 670 nm, respectively.

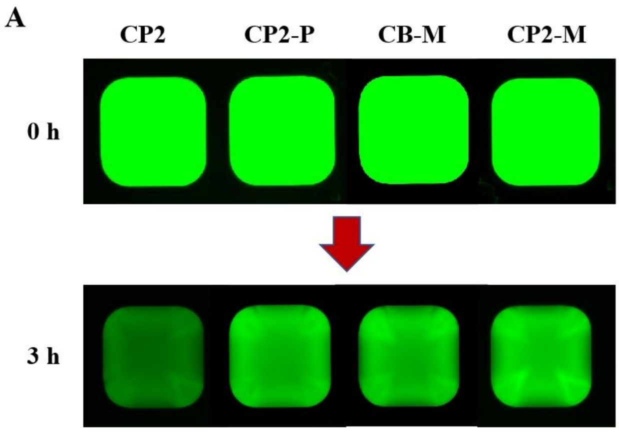

B

Irradiation time	0 h	3 h
CP2	246.3(100%)	132.6(54%)
CP2-P	394.0(100%)	316.7(80%)
CB-M	401.9(100%)	309.5(77%)
CP2-M	428.5(100%)	338.7(79%)

Figure S5. **Photophysical stability of the probes.** (**A**) Imaging phantoms containing 120 mL water droplets containing CP2, CP2-P, CB-M, or CP2-M (2 µM in PBS buffer, pH 6.0) were irradiated with a fluorescent stereomicroscope's LED for 3 hours (λex=740 nm, λem =780-820 nm, 6.0 W/cm²). (**B**)The mean fluorescence intensity (MFI) values for the fluorescence images are listed (N=3).

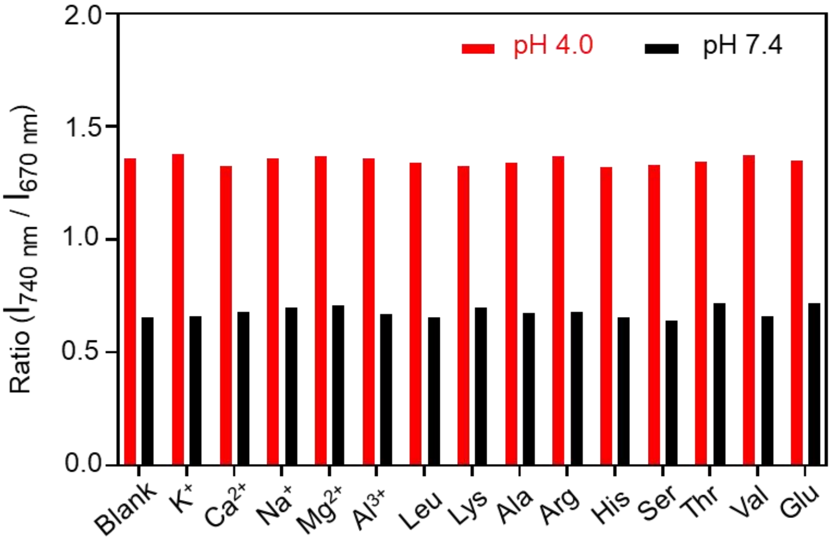

Figure S6. CP2-M showed selectivity for sensing pH. Fluorescence intensity ratio (I_{740}/I_{670} nm) of CP2-M (5.0 µM) in the presence of physiological ion or amino acid in PBS with pH 4.0 (red) and pH 7.4 (black).

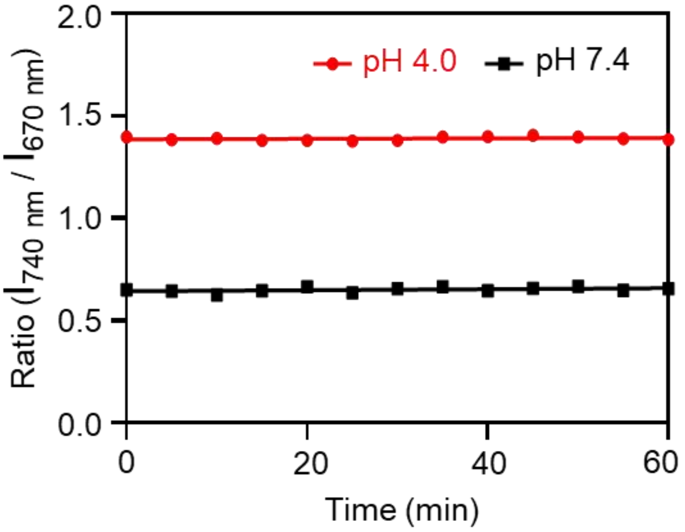

Figure S7. CP2-M shows photophysical stability in aqueous solutions. Time course of the fluorescence intensity ratios (I_{740}/I_{670} nm) of CP2-M (5.0 µM) in PBS buffer solutions with pH 4.0 and pH 7.4.

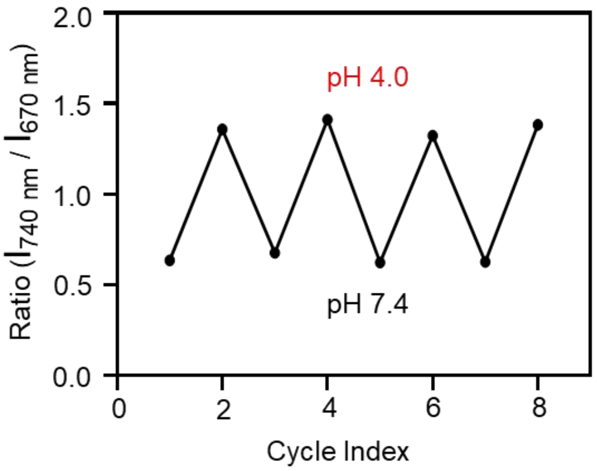

Figure S8. CP2-M shows reversible pH responsiveness. Fluorescence reversibility of CP2-M (5.0 µM) in PBS between pH 4.0 and pH 7.4.

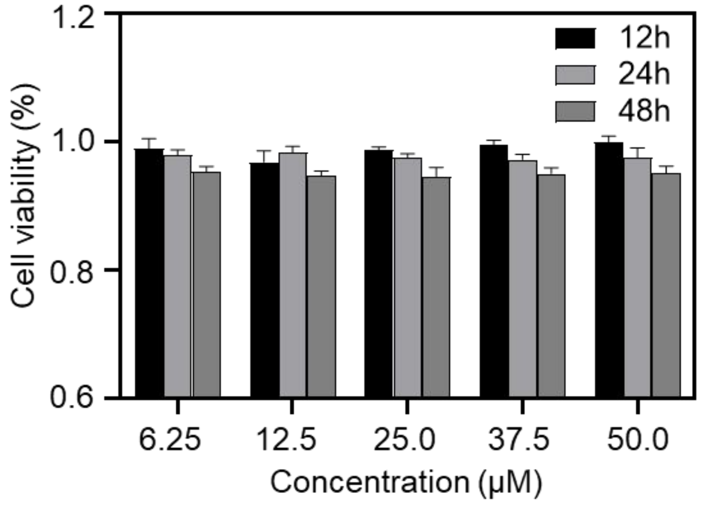

Figure S9. CP2-M shows minimized cytotoxicity. Viabilities of M2-TAMs after treatment of CP2-M for 12 h, 24 h, and 48 h with concentrations ranged from 6.25 to 50.0 μM. Cell viabilities were determined by CCK-8 assay. Data are presented as the mean ± s.d. for three replicates.

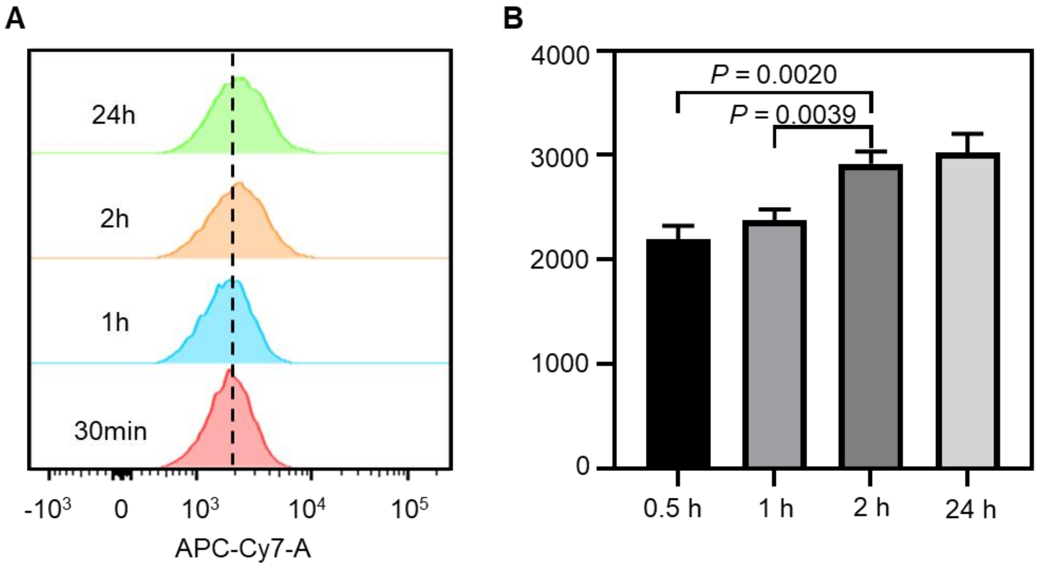

Figure S10. Time-dependent cellular uptake of CP2-M in M2-Mφ. Flow cytometric analysis of M2-Mφ (5.0 μM) treated with CP2-M for 0.5h, 1h, 2h, 24h.

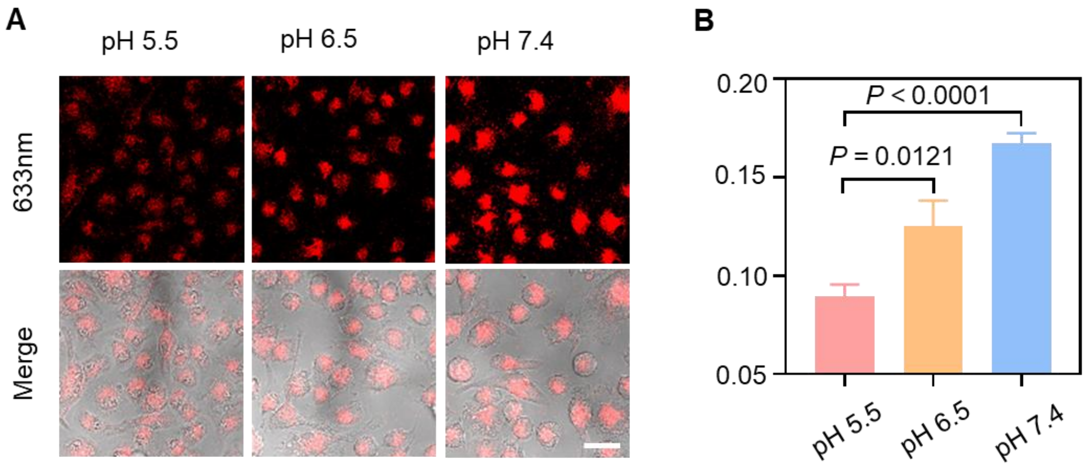

Figure S11. CP2-M respond to pH changes in vitro. （**A**） Confocal microscopic images of M2-Mφ (25.0 μM) treated with media with different pH values. Scale bar: 30 μm. （**B**） the average signal intensity in 633 nm channel at each of the following pH values: 7.4, 6.5, and 5.5.

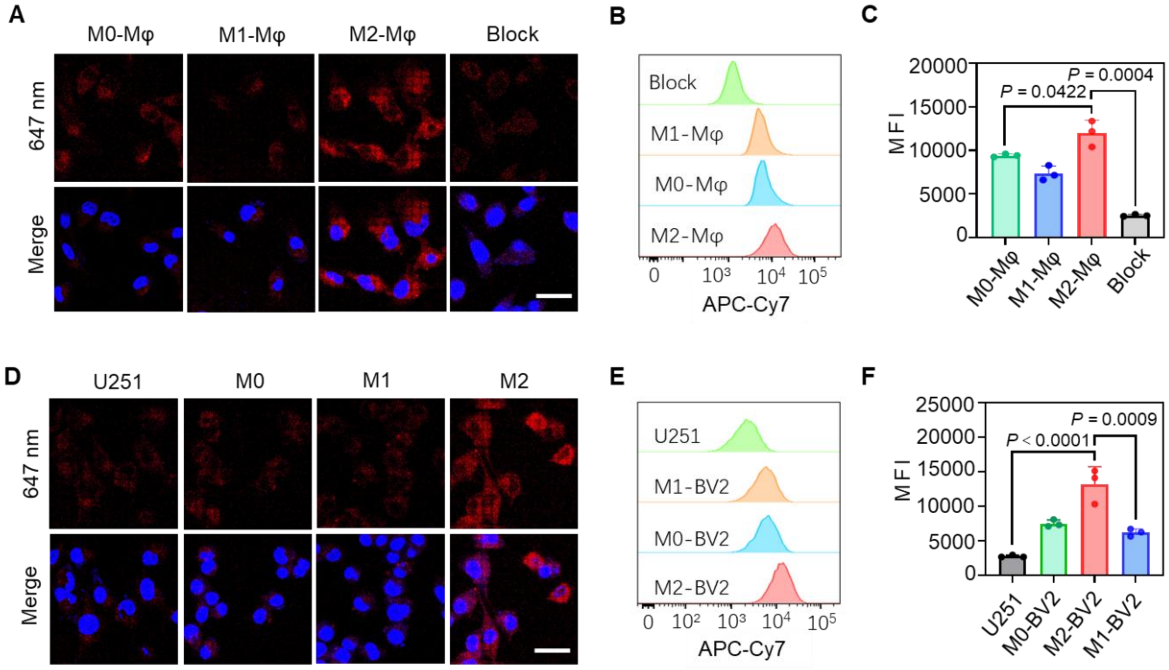

Figure S12. Macrophage and microglia show similar phenotype dependent CP2-M uptake behaviors. (A, D) Confocal fluorescence images of live U251, microglia (M0, M1 and M2) or macrophages (M0, M1 and M2) treated with CP2-M (25.0 μM) for 1 h, scale bar: 30 μm; Flow cytometric analysis of the CP2-M endocytosed by macrophages (B) or microglia (E) of different phenotypes. (C, F) Quantification of the intracellular mean fluorescence intensities (MFI) demonstrated in the panel B or E.

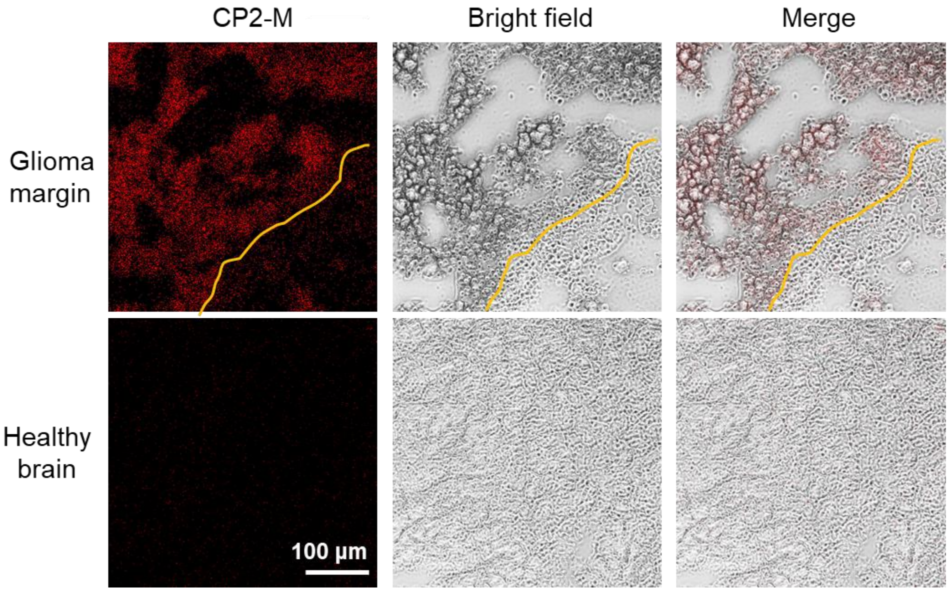

Figure S13. Representative fluorescence (CP2-M) imaging of brain sections from healthy mice and glioma-bearing mice allograft at 4 hours post administration of CP2-M via the tail vein (5.0 μmol/kg/rat). The yellow solid lines indicate the tumor margins, scale bar: 100 μm.

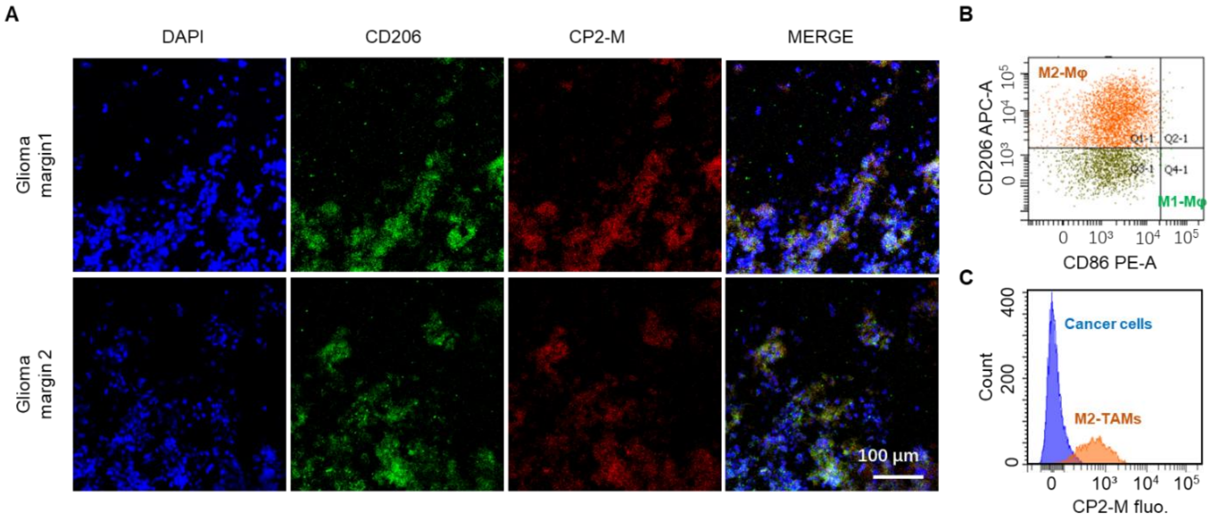

Figure S14. CP2-M visualizes glioma margins via hierarchical strategy. (A) Fluorescence images of brain sections from glioma-bearing mice at 4 h post intravenous administration of CP2-M. CD206 (green) and CP2-M (red). scale bar: 100 μm. (B) Flow cytometric analysis of immunostimulatory $CD206^{+}$ $CD86^{-}$ macrophages (M2-TAMs) and CD206- CD86+ macrophages (M1-TAMs) from glioma marginal tissue. (C) The content of CP2-M uptaken by various cells (cancer cells, M1-TAMs, and M2-TAMs) at the glioma margin.

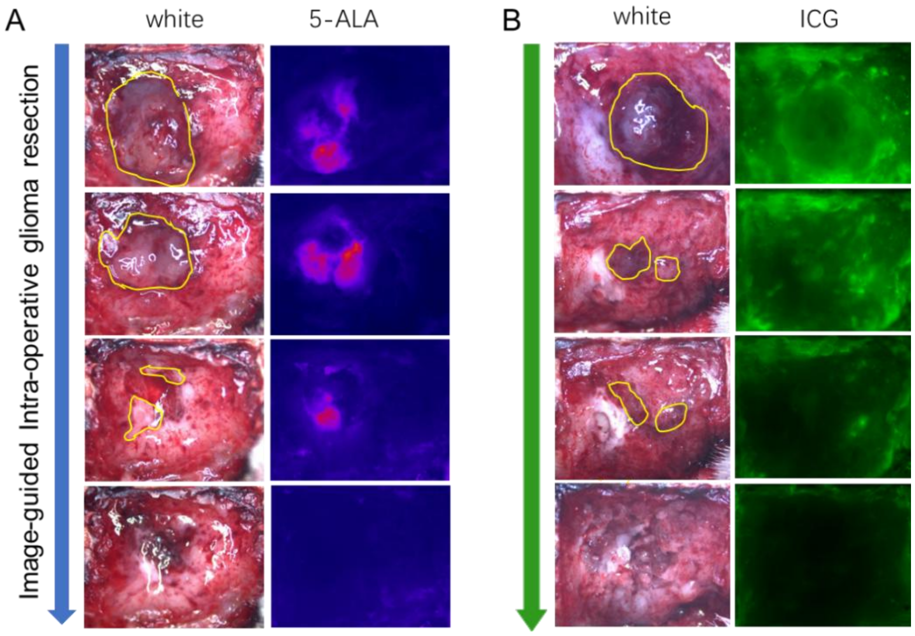

Figure S15. Image-guided surgery by using clinically approved fluorescence probes including 5-ALA and ICG in live rat models bearing C6 GBM allografts.

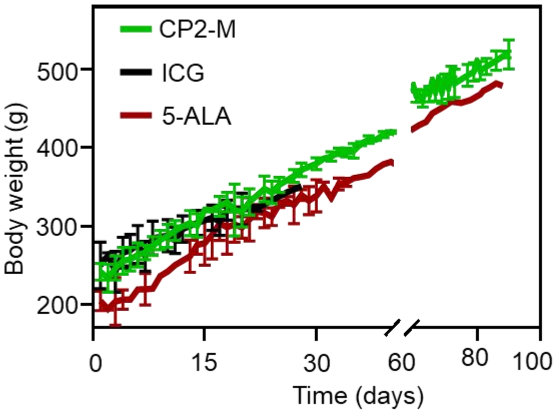

Figure S16. Body weight of rat models after different treatments (n = 5 rats/group).

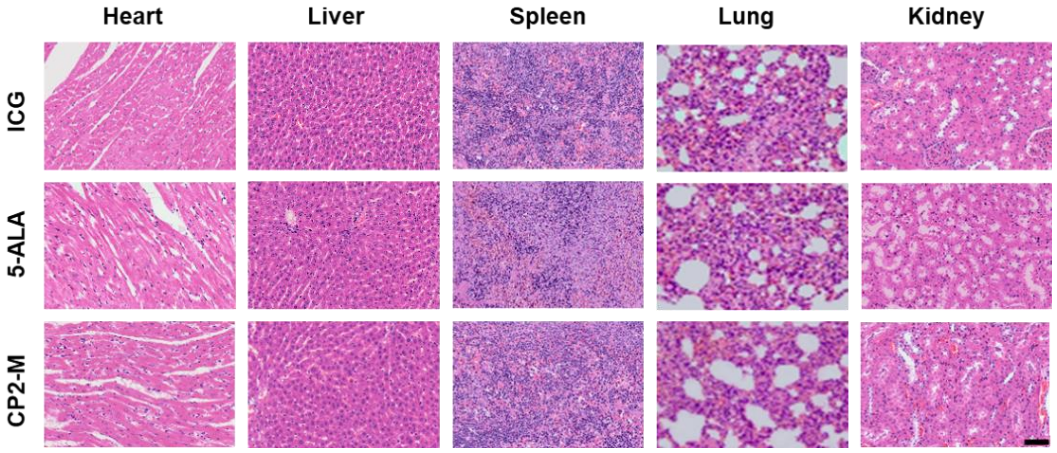

Figure S17. CP2-M shows minimized toxicity for the major organs of rats. H&E staining of major organs (heart, liver, spleen, lung, kidney) from healthy rat treated with ICG, 5-ALA, or CP2-M (25 μmol kg^{-1}). The organs were harvested at 14 days after administration of ICG, 5-ALA, or CP2-M. No obvious abnormities or lesions were observed. Scale bar: 50 μm.

General synthesis procedure and the chemical characterization of CP2-M (Scheme 1.):

Synthesis of compound C1. C1 was prepared following the previous published procedures.[29]

Synthesis of compound C2. A mixture of 2,3,3-trimethylbenzoindolenine (10.0 g, 62.8 mmol) and methyl 3-bromopropanoate (11.8 g, 70.9 mmol) in anhydrous acetonitrile (80 mL) was heated to reflux for 12 h. After cooling to room temperature, the resulting solid was washed with diethyl ether until colorless, and dried in vacuo for 24 h to give compound C2 (13.7 g, 66.9%). C2 was directly used in the next step without purification. ^{1}H NMR (400 MHz, DMSO-d_6, δ): 7.89 – 7.93 (d, J = 3.6 Hz, 1H), 7.74 – 7.80 (m, 1H), 7.51 – 7.59 (m, 2H), 4.56 – 4.65 (t, J = 6.7 Hz, 2H), 3.54 (s, 3H), 2.96 – 3.05 (t, J = 6.5 Hz, 2H), 2.79 (s, 3H), 1.46 (s, 6H). ^{13}C NMR (151 MHz, DMSO-d_6, δ): 198.17, 170.50, 141.75, 140.86, 129.34, 128.89, 123.48, 115.55, 54.30, 51.86, 43.36, 30.85, 21.91, 14.42. HRMS (ESI) m/z: $[M]^+$ calcd for $C_{15}H_{20}NO_2^+$: 246.1494; found: 246.1490.

Synthesis of compound C3. A mixture of C1 (1.0 g, 5.8 mmol), C2 (3.6 g, 11 mmol), and sodium acetate (1.0 g, 12.2 mmol) in acetic anhydride (20 mL) was heated at 80 °C for 2 h and then cooled to room temperature. The reaction solution was added dropwise to diethyl ether for precipitation, and the precipitated solid was washed with diethyl ether (60 mL × 2). Purification by silica gel column chromatography [CH_2Cl_2:MeOH = 10:1 to 5:1, V:V] provided pure compound C3 (2.6 g, 63.4%) as a dark green solid. ^{1}H NMR (400 MHz, DMSO-d_6, δ): 8.37 – 8.44 (d, J = 13.9 Hz, 2H), 7.46 – 7.52 (d, J = 7.2 Hz, 2H), 7.42 – 7.19 (m, 6H), 6.32 – 6.40 (d, J = 14.0 Hz, 2H), 4.45 – 4.48 (d, J = 6.2 Hz, 4H), 3.55 – 3.60 (m, 6H), 2.82 – 2.88 (t, J = 6.4 Hz, 4H), 2.69 – 2.75 (m, 4H), 1.92 – 1.96 (m, 2H), 1.69 (s, 12H). ^{13}C NMR (151 MHz, MeOD, δ): 174.42, 172.72, 151.45, 145.75, 143.15, 142.53, 129.87, 128.63, 126.65, 123.56, 112.26, 102.88, 52.63, 50.68, 41.21, 32.62, 28.34, 27.36, 22.11. HRMS (ESI) m/z: $[M]^+$ calcd for $C_{38}H_{44}ClN_2O_4^+$: 627.2990; found: 627.2978.

Synthesis of compound C4. Compound C3 (1.0 g, 1.4 mmol) and N-ethylpiperazine (0.48 g, 4.2 mmol) were dissolved in anhydrous acetonitrile (30 mL), and stirred at 25 °C for 6 h. The solvent was removed under reduced pressure, then the crude product was purified by column chromatography on silica gel [CH_2Cl_2:MeOH = 10:1 to 4:1, V:V] to afford a blue solid C4 (0.7 g, 63.2%). ^{1}H NMR (400 MHz, MeOD, δ): 7.63 – 7.70 (d, J = 13.2 Hz, 2H), 7.35 – 7.42 (d, J = 7.1 Hz, 2H), 7.26 – 7.34 (t, J = 7.5 Hz, 2H), 7.08 – 7.15 (m, J = 7.3 Hz, 4H), 5.93 – 6.02 (d, J = 13.5 Hz, 2H), 4.25 – 4.33 (t, J = 5.6 Hz, 4H), 3.80 – 3.85 (m, 4H), 3.53 – 3.58 (m, 6H), 2.72 – 2.79 (m, 8H), 2.48 – 2.55 (m, J = 6.0 Hz, 6H), 1.76 – 1.83 (d, J = 6.6 Hz, 2H), 1.63 (s, 12H), 1.18 (s, 3H). ^{13}C NMR (151 MHz, MeOD, δ): 175.81, 173.05, 170.14, 143.67,

142.77, 141.53, 129.52, 125.71, 124.71, 123.22, 110.61, 97.21, 55.86, 55.40, 53.48, 52.57, 40.26, 34.30, 32.33, 29.31, 25.71, 23.00, 11.87. HRMS (ESI) m/z: $[M]^+$ calcd for $C_{44}H_{57}N4O_4^+$: 705.4374; found: 705.4368.

Synthesis of compound CP2. Compound 4 (0.5 g, 0.64 mmol) was dissolved in methanol (30 mL), followed by addition of aqueous potassium hydroxide (67 mM, 20 mL) to the above solution at 0 °C and stirred at room temperature for 4 h. The methanol was removed and the saturated aqueous ammonium chloride solution was added for adjusting to pH 7, extracted with CH_2Cl_2 (3×). The solvent was removed under reduced pressure, then the crude product was purified by column chromatography on silica gel [CH_2Cl_2:MeOH = 10:1 to 3:1, V:V] to afford a blue solid CP2 (0.23 g, 47.3%). ^{1}H NMR (400 MHz, $CDCl_3$, δ): 7.70 – 7.75 (d, *J* = 13.4 Hz, 2H), 7.35 – 7.41 (d, *J* = 7.3 Hz, 2H), 7.27 – 7.34 (t, *J* = 7.4 Hz, 2H), 7.16 – 7.23 (d, *J* = 7.8 Hz, 2H), 7.07 – 7.15 (t, *J* = 7.2 Hz, 2H), 6.05 – 6.13 (d, *J* = 13.4 Hz, 2H), 4.24 (t, *J* = 7.0 Hz, 4H), 3.75 (s, 4H), 3.32 (s, 1H), 3.11 (d, *J* = 4.0 Hz, 4H), 2.87 – 2.76 (m, 4H), 2.66 (d, *J* = 5.8 Hz, 6H), 1.85 – 1.75 (m, 2H), 1.65 (s, 12H), 1.09 (t, *J* = 7.1 Hz, 3H). ^{13}C NMR (151 MHz, MeOD, δ): 178.08, 174.08, 170.56, 143.93, 143.06, 141.79, 129.56, 126.27, 124.77, 123.09, 111.01, 97.94, 55.37, 53.12, 50.99, 44.63, 42.12, 35.60, 29.20, 25.94, 23.10, 11.71. HRMS (ESI) m/z: $[M]^+$ calcd for $C_{42}H_{53}N_4O_4^+$: 677.4061; found: 677.4070.

Synthesis of compound CP2-M. The compounds CP2 (100 mg, 0.13 mmol), NHS (76 mg, 0.66 mmol), DCC (136 mg, 0.66 mmol) were dissolved in 20 mL of acetonitrile and reacted at 25 °C for 0.5 h. PEG_{2k}-mannose (500 mg, 0.25 mmol) was dissolved in aqueous solution (20 mL) at pH 8, and then added dropwise to the above reaction solution in batches for further reaction. The reaction was kept in dark for 2 h, and the product was further purified via vacuum distillation, dialysis, lyophilization and silica gel column chromatography (CH_2Cl_2:CH_3OH= 10:1 to 3:1, V:V) to obtain pure CP2-M of 162 mg with a yield of 46.7%. ^{1}H NMR (400 MHz , MeOD, δ): 8.54 – 8.51 (t , 1H) , 7.71 (d , *J* = 14.4 Hz , 2H) , 7.47 (d , *J* = 7.5 Hz , 2H) , 7.41-7.37 (t , 2H) , 7.23 (d , *J* = 7.0 Hz , 2H) , 7.19 – 7.14 (t , 2H) , 6.07 (d , *J* = 13.5 Hz , 2H) , 4.34-4.31 (t , 4H) , 3.84-3.83 (m , 9H) , 3.59 (s , 187H) , 3.18-3.16 (m , 8H) , 2.69-2.66 (t , 4H) , 2.51-2.48 (m , 6H) , 1.95 (s , 12H) , 1.81 – 1.75 (m , 2H) , 1.12 (s , 3H). HRMS (ESI) m/z: $[M]^+$ found for 2375.3876.

Synthesis of compound C5. Compound C3 (0.5 g, 0.71 mmol) was dissolved in methanol (30 mL), followed by addition of aqueous potassium hydroxide (74 mM, 20 mL) to the above solution at 0 °C and stirred at room temperature for 4 h. The methanol was removed and the saturated aqueous ammonium chloride solution was added for adjusting to pH 7, extracted with CH_2Cl_2 (3×). The solvent was removed under reduced pressure, and the obtained compound C5 were used directly in the next step without purification.

Synthesis of compound CB-M. The compounds C5 (100 mg, 0.15 mmol), NHS (84.6 mg, 0.74 mmol), DCC (151.7 mg, 0.74 mmol) were dissolved in 20 mL of acetonitrile and reacted at 25 °C for 0.5 h. PEG_{2k}-mannose (500 mg, 0.25 mmol) was dissolved in aqueous solution (20 mL) at pH 8, and then added dropwise to the above reaction solution in batches for further reaction. The reaction was kept in dark for 2 h, and the product was further purified via vacuum distillation, dialysis, lyophilization and silica gel column chromatography (CH_2Cl_2:CH_3OH= 10:1 to 3:1, V:V) to obtain pure CP2-M of 191 mg with a yield of 48.2%.^{1}H NMR (400 MHz , MeOD, δ): 8.48-8.43 (d, *J* = 14.1 Hz, 2H), 7.67 - 7.19 (m, 8H), 6.40 (d, *J* = 14.1 Hz, 2H), 4.53-4.48 (t, *J* = 6.7 Hz, 4H), 3.87 – 3.74 (m, 1H), 3.63 (m, *J* = 3.5 Hz, 187H), 3.33 (m, *J* = 15.9 Hz, 10H), 2.89 (t, *J* = 6.7 Hz, 4H), 2.77 (t, *J* = 6.1 Hz, 4H), 2.00-1.96 (t, 2H), 1.74 (s, 12H), 1.21-1.14 (t, *J* = 6.8 Hz, 3H).

NMR and MS spectrum data

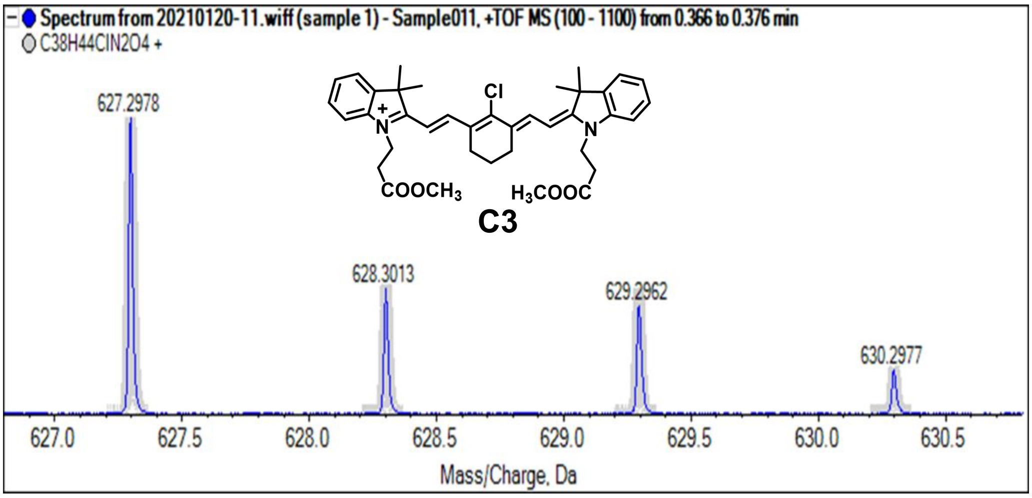

Data S1. High-resolution mass spectrometry (HR-MS) of compound C3.

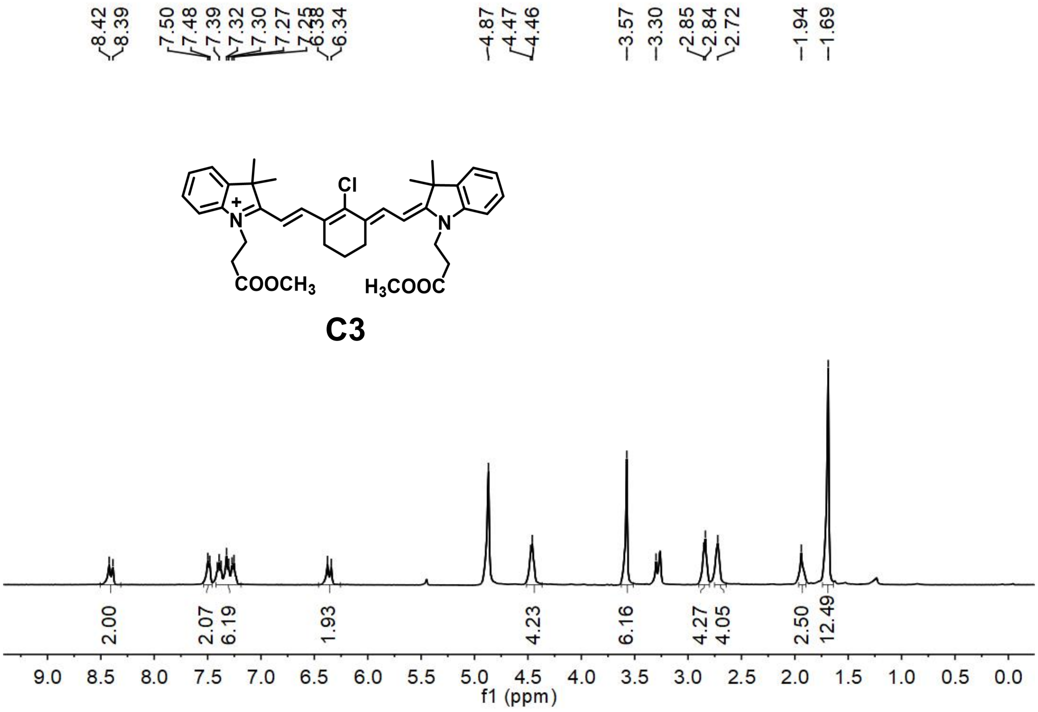

Data S2. ^{1}H NMR spectrum of compound C3 in MeOD.

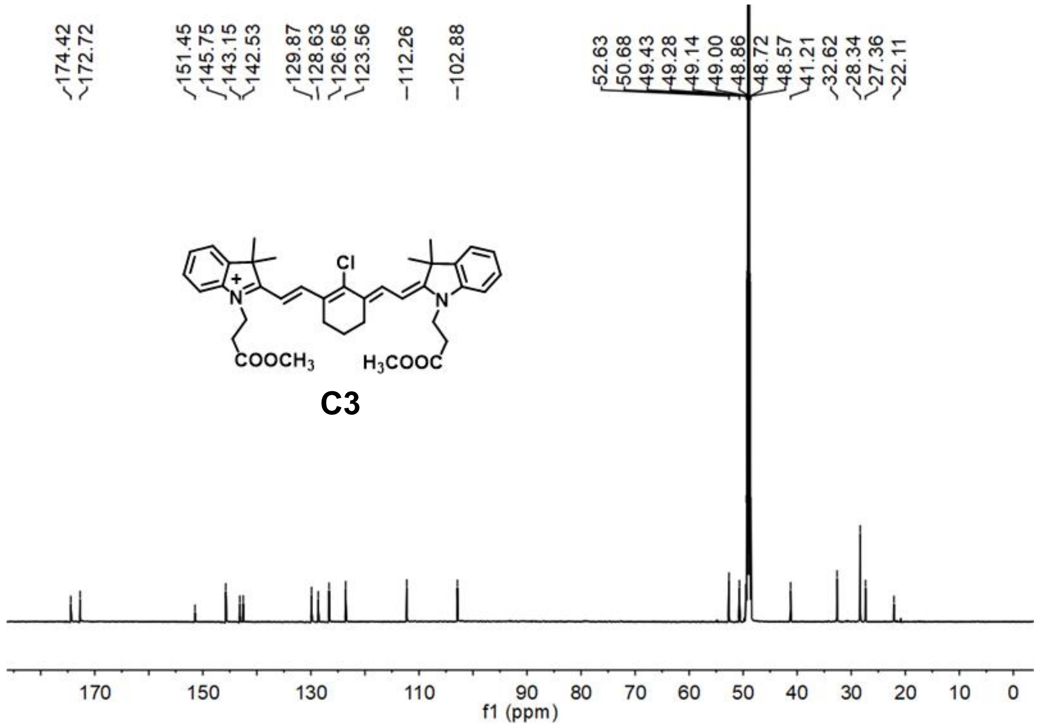

Data S3. ^{13}C NMR spectrum of compound C3 in MeOD.

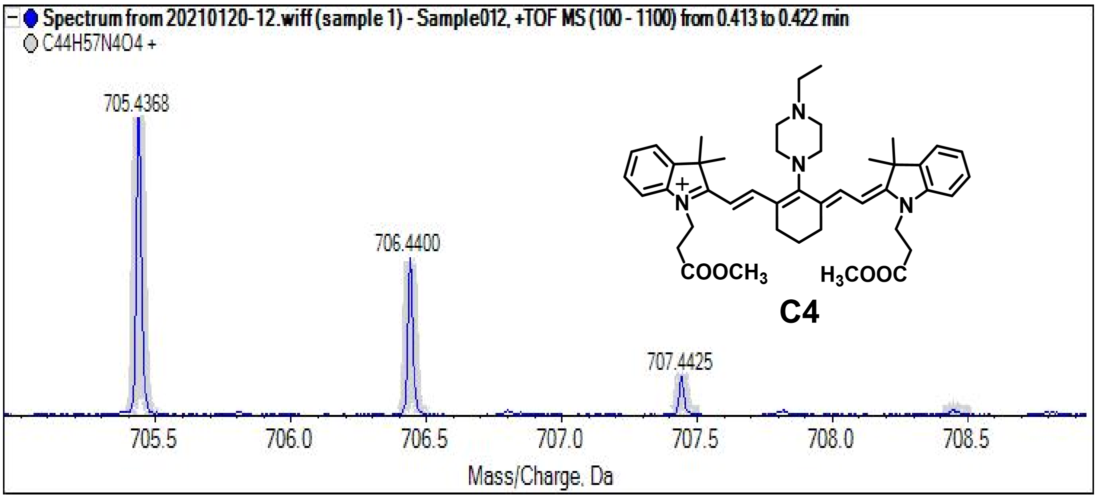

Data S4. HR-MS spectrum of compound C4.

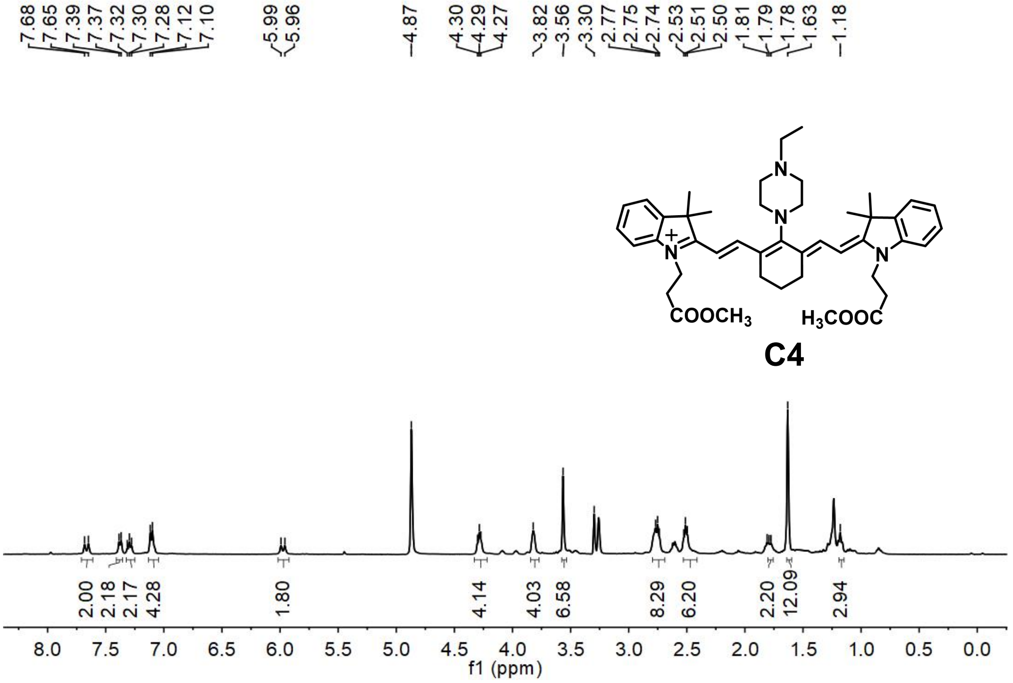

Data S5. ^{1}H NMR spectrum of compound C4 in MeOD.

C4

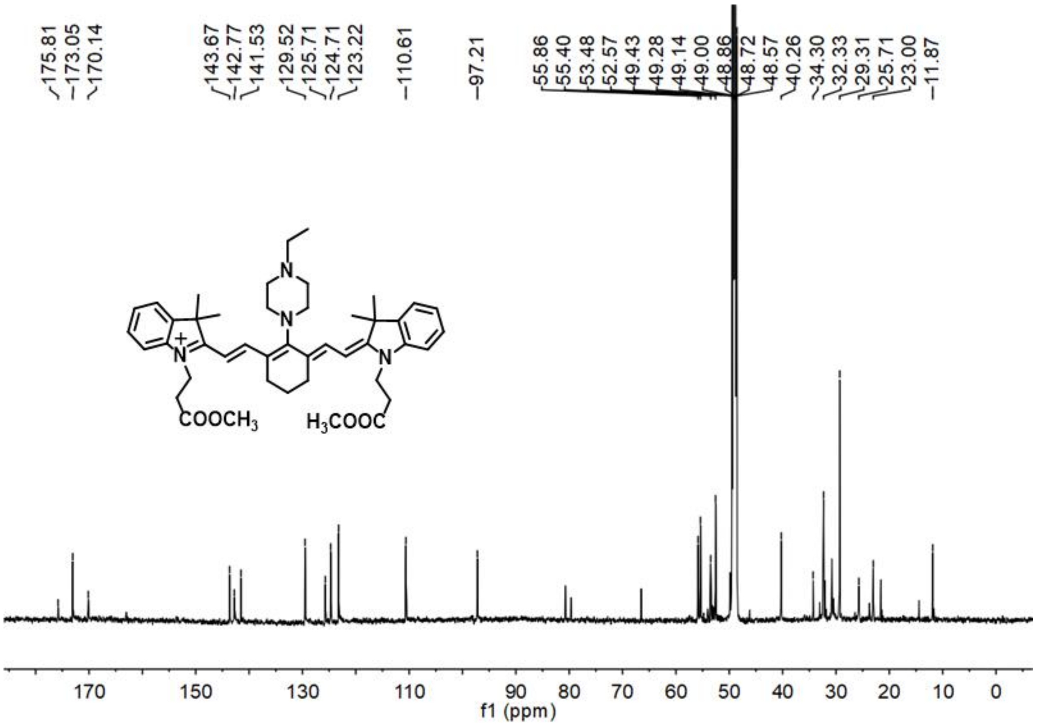

Data S6. ^{13}C NMR spectrum of compound C4 in MeOD.

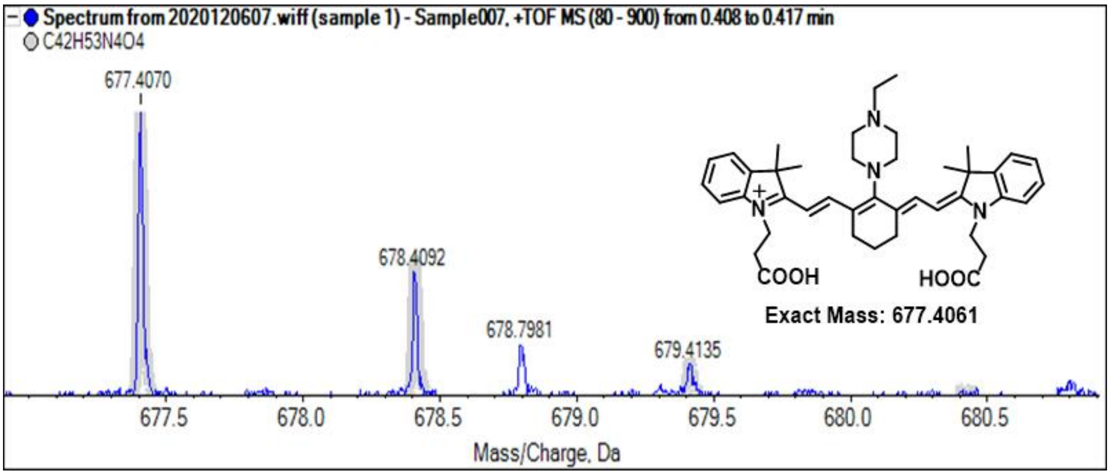

Data S7. HR-MS spectrum of compound CP2.

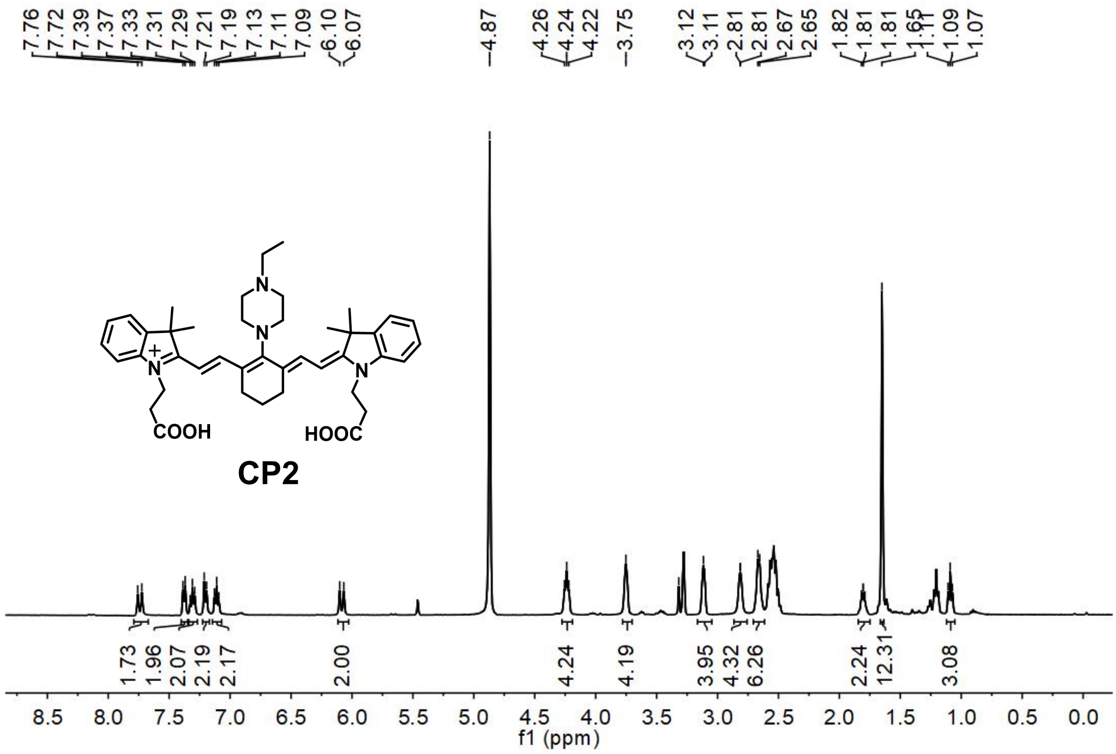

Data S8. ^{1}H NMR spectrum of compound CP2 in MeOD.

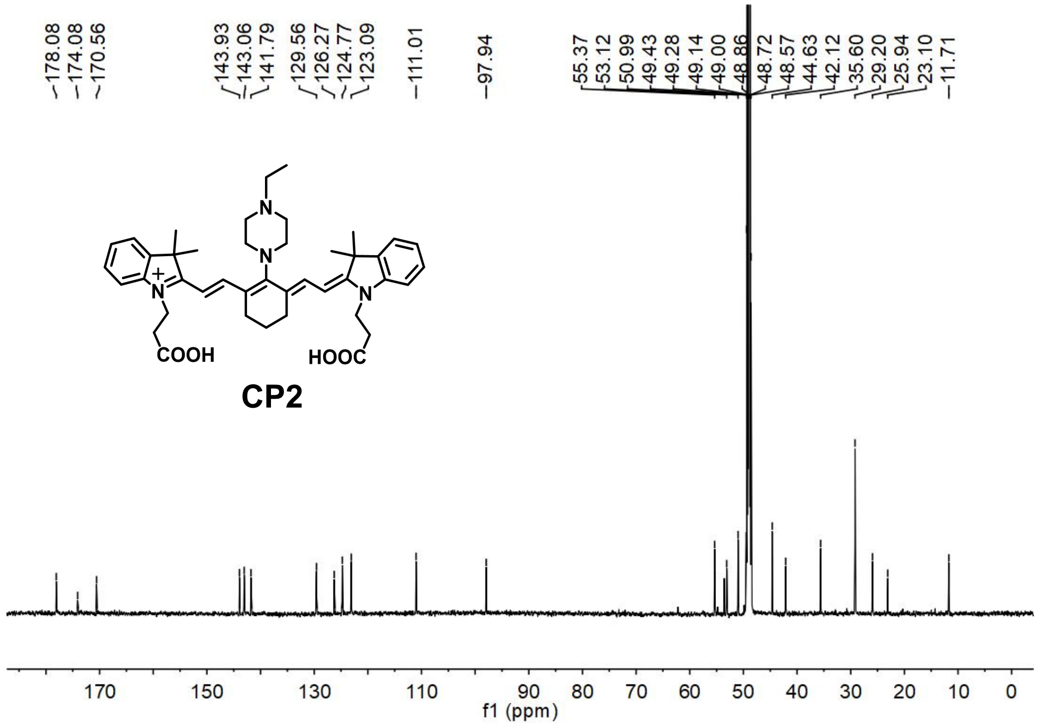

Data S9. ^{13}C NMR spectrum of compound CP2 in MeOD.

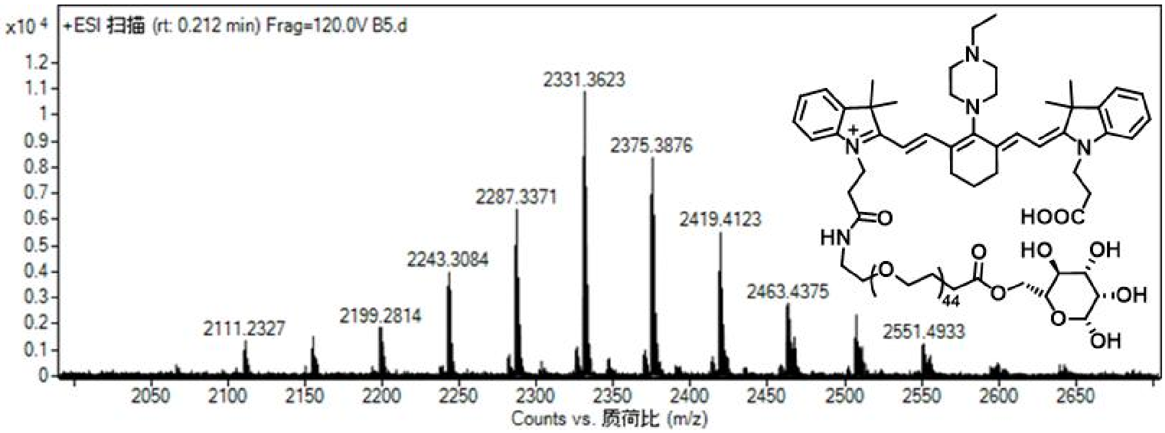

Data S10. Mass spectra of aiming probe CP2-M.

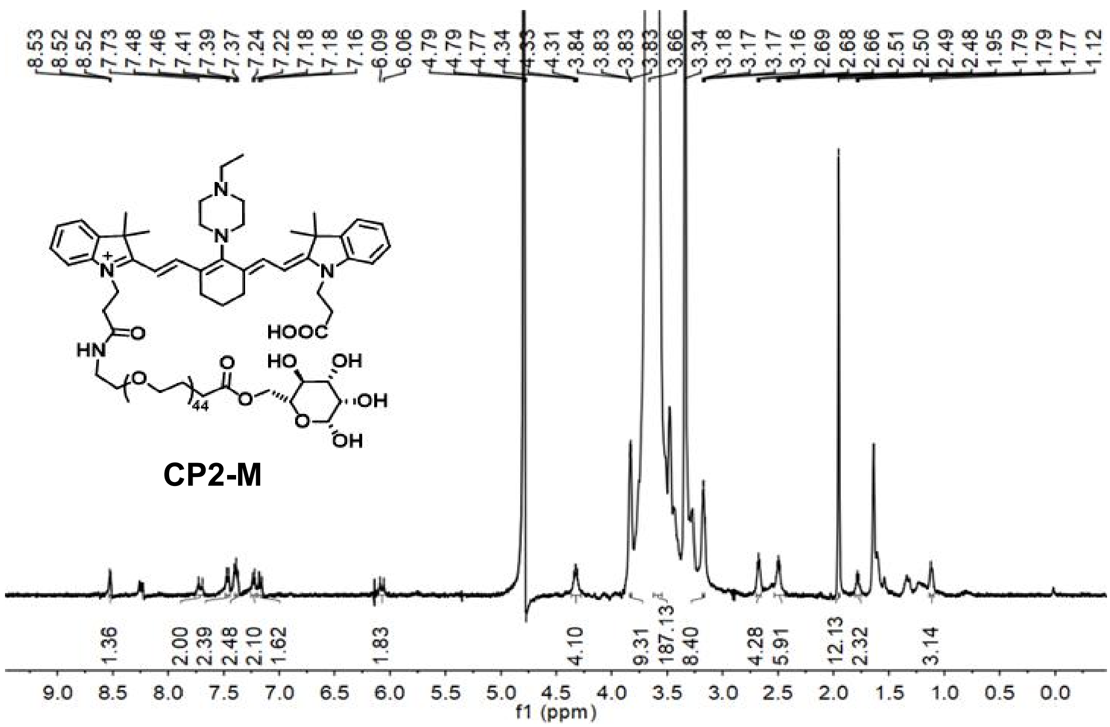

Data S11. ^{1}H NMR spectrum of compound CP2-M in MeOD.

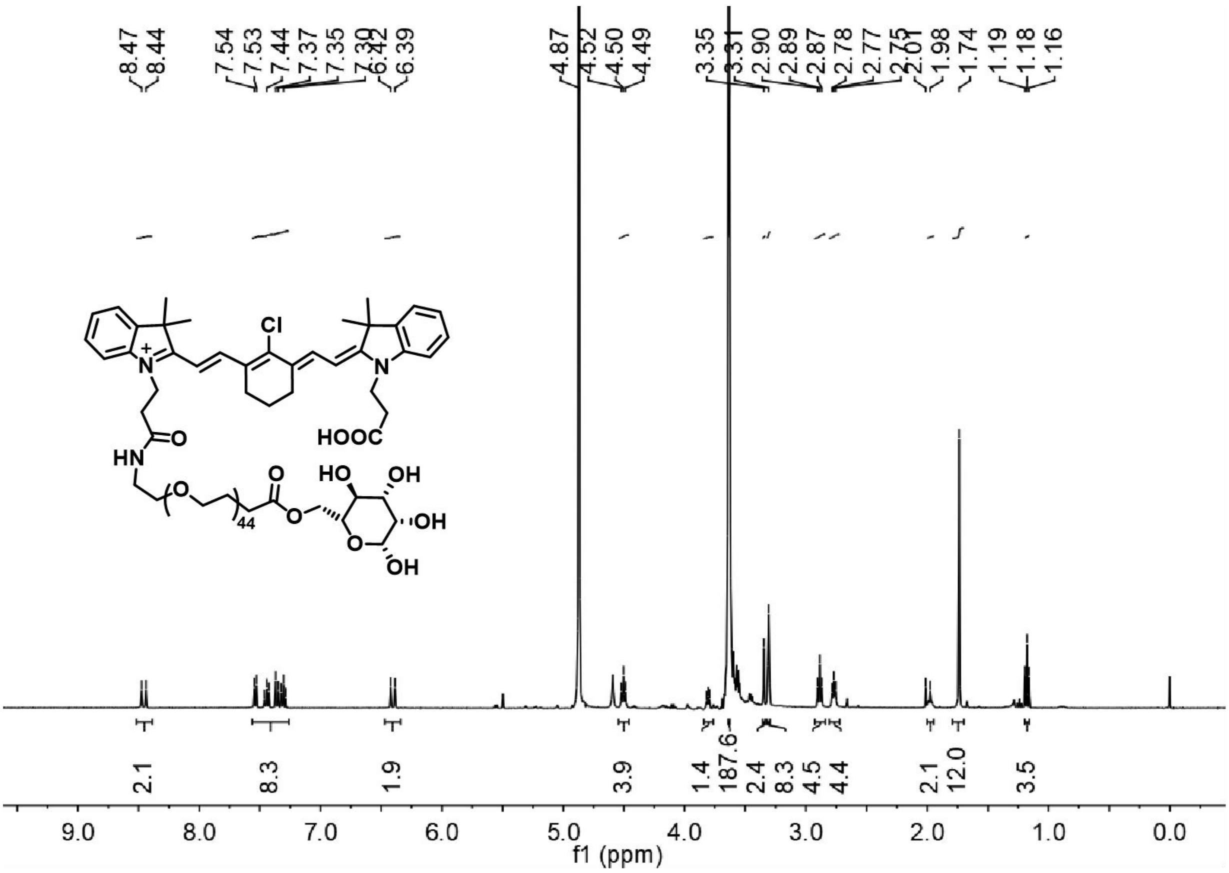

Data S12. ^{1}H NMR spectrum of compound CB-M in MeOD.
